# Pyroptosis correlates with tumor immunity and prognosis

**DOI:** 10.1038/s42003-022-03806-x

**Published:** 2022-09-06

**Authors:** Xiaoying Lou, Kexin Li, Benheng Qian, Yiling Li, Donghong Zhang, Wei Cui

**Affiliations:** 1 State Key Laboratory of Molecular Oncology, Department of Clinical Laboratory, National Cancer Center/National Clinical Research Center for Cancer/Cancer Hospital, Chinese Academy of Medical Sciences and Peking Union Medical College, Beijing, 100021 China; 2 Department of Cardiology, The Second Affiliated Hospital of Wenzhou Medical University, 109 Xueyuan Road, Wenzhou, 325027 Zhejiang PR China; 3 Center for Molecular and Translational Medicine, Research Science Center, Georgia State University, Atlanta, GA 30303 USA; 4 Department of Clinical Laboratory, National Cancer Center/National Clinical Research Center for Cancer/Cancer Hospital, Chinese Academy of Medical Sciences and Peking Union Medical College, Key Practice of Laboratory Medicine in Qinghai Province, Beijing, 100021 China

**Keywords:** Immunosurveillance, Cell death and immune response, Data mining, Predictive markers

## Abstract

Pyroptosis, as a proinflammatory form of regulated cell death, plays an important role in multiple cancers. However, the diagnostic and prognostic values of pyroptosis and its interaction with tumor immunity in pan-cancer are still unclear. Here, we show an elevated general expression of 17 pyroptosis-associated genes of tumor patients with high-immune-activity and a reduced pyroptosis in low-immune-activity tumors. Moreover, pyroptosis is positively correlated with immune infiltration and immune-related signatures across 30 types of cancer. Furthermore, our experimental data suggest that pyroptosis directly modulate the expression of immune checkpoint molecules and cytokines. We generate a pyroptosis score model as a potential independent prognostic indicator in melanoma patients. Interestingly, 3 of pyroptosis-associated genes including CASP1, CASP4 and PYCARD, can predict the effectiveness of anti-PD-1 immunotherapy for patients with melanoma. Our study demonstrates that pyroptosis correlates with tumor immunity and prognosis, might be used as a potential target for immune therapy.

## Abstract (web summary)

A pan-cancer analysis demonstrates that pyroptosis is involved in tumor development, prognosis and immunotherapy through the promotion of tumor immunity.

## Introduction

Pyroptosis, a proinflammatory form of regulated cell death (RCD), is characterized by cellular swelling, lysis, and the release of proinflammatory cytokines. It was first observed in macrophages treated with anthrax lethal toxin in 1986 and then identified in myeloid cells infected with pathogenic bacteria, which misclassifies pyroptosis as apoptosis and delays the recognition of this type of cell death as pyroptosis for nearly two decades^1–4^. Subsequently, Cookson et al. proposed the term “pyroptosis” to define this type of cell death and pointed out that it is an alternative pathway that has evolved from a simple and quiet method of death into a visible proinflammatory response of cell death^5^.

Until 2015, the key molecular mechanism of pyroptosis has been elucidated for the first time. Gasdermin D (GSDMD), a key member of gasdermin family, was the executor of pyroptosis and could be cleaved and activated by caspase-1/4/5/11^6,7^. The N-terminal fragment of GSDMD oligomerizes in membranes to form a pore that leads to the occurrence of pyroptosis^8,9^. Subsequently, pyroptosis was defined as gasdermin-mediated programmed necrosis^10^. Recent studies have shown that GSDME, another member of the gasdermin family, could be cleaved and activated by caspase-3, leading to the transition from apoptosis to pyroptosis^11,12^. In 2018, the Nomenclature Committee on Cell Death (NCCD) redefined the concept of “pyroptosis”, as a type of RCD that is critically dependent on members of the gasdermin (GSDM) family to form plasma membrane pores, usually (but not always) as the result of inflammatory caspase activation^13^.

The process of pyroptosis can be divided into three phases includes an initiation phase, activation phase, and effector phase^14^. The initiation phase involves the activation of upstream proteins of the GSDM family, such as inflammasomes and chemotherapy drugs^15–17^. The inflammasome was assembled in the stimulated cells to recruit and activate members of the caspase family, such as caspase-1/4/5/11^18–20^. Subsequently, members of the gasdermin family, such as GSDMD, GSDME, and GSDMB, were cleaved, and then their N-terminal was exposed and allowed to bind to phospholipids of the cell membrane, during the activation phase^21^. Moreover, the precursors of the proinflammatory cytokines (pro-IL-1β and pro-IL-18) were cleaved by activated caspase-1^22^. Next, the effector phase involved the rupture of the cell membrane and the release of activated inflammatory factors (IL-1β and IL-18), which triggered a series of inflammatory cascade amplification reactions^14,23^.

Due to the proinflammatory properties of pyroptosis, it produced a dual effect on the tumors^24^. As a form of programmed cell death, pyroptosis could inhibit the growth of tumor cells; while, the inflammatory response induced by pyroptosis provided a suitable microenvironment for tumor cell survival^25^. Interestingly, several studies reported that pyroptosis could induce an anti-tumor immune response^26–28^. GSDME could mediate cancer cell pyroptosis by granzyme B released by killer cells, which increases the infiltration of killer lymphocytes^26^. Meanwhile, GSDME could enhance the infiltration of CD4+/CD8+ T cells based on a new bio-orthogonal system. Thus, GSDME induced tumor clearance is immune-dependent^28^. Subsequently, another study showed that granzyme A released from cytotoxic NK and T lymphocytes could cleave GSDMB, thereby triggering the prolapse of target cells and leading to tumor clearance^27^. Therefore, an understanding of the interaction between pyroptosis and immune response may provide a potential strategy for immune checkpoint blockade (ICB) therapy.

In this study, we systematically analyzed the expression levels of the pyroptosis-associated genes (PAGs) and their correlation with genetic alterations, including mutations and copy number variations in pan-cancer. To further understand the mechanism of pyroptosis-related anti-tumor immunity, we explored the relationship between the general expression pattern of pyroptosis and immunocompetence with bioinformatical analysis and experiments. Next, we investigated the prognostic function and the predictive effect on the anti-programmed cell death protein 1 (PD-1) treatment of PAGs in patients with skin cutaneous melanoma (SKCM). Our pan-cancer analysis demonstrated that pyroptosis is involved in tumor development, prognosis, and ICB therapy might through the modulation of tumor immune pathway.

## Results

### The landscape of the PAGs across cancer types

**Fig. 1:**
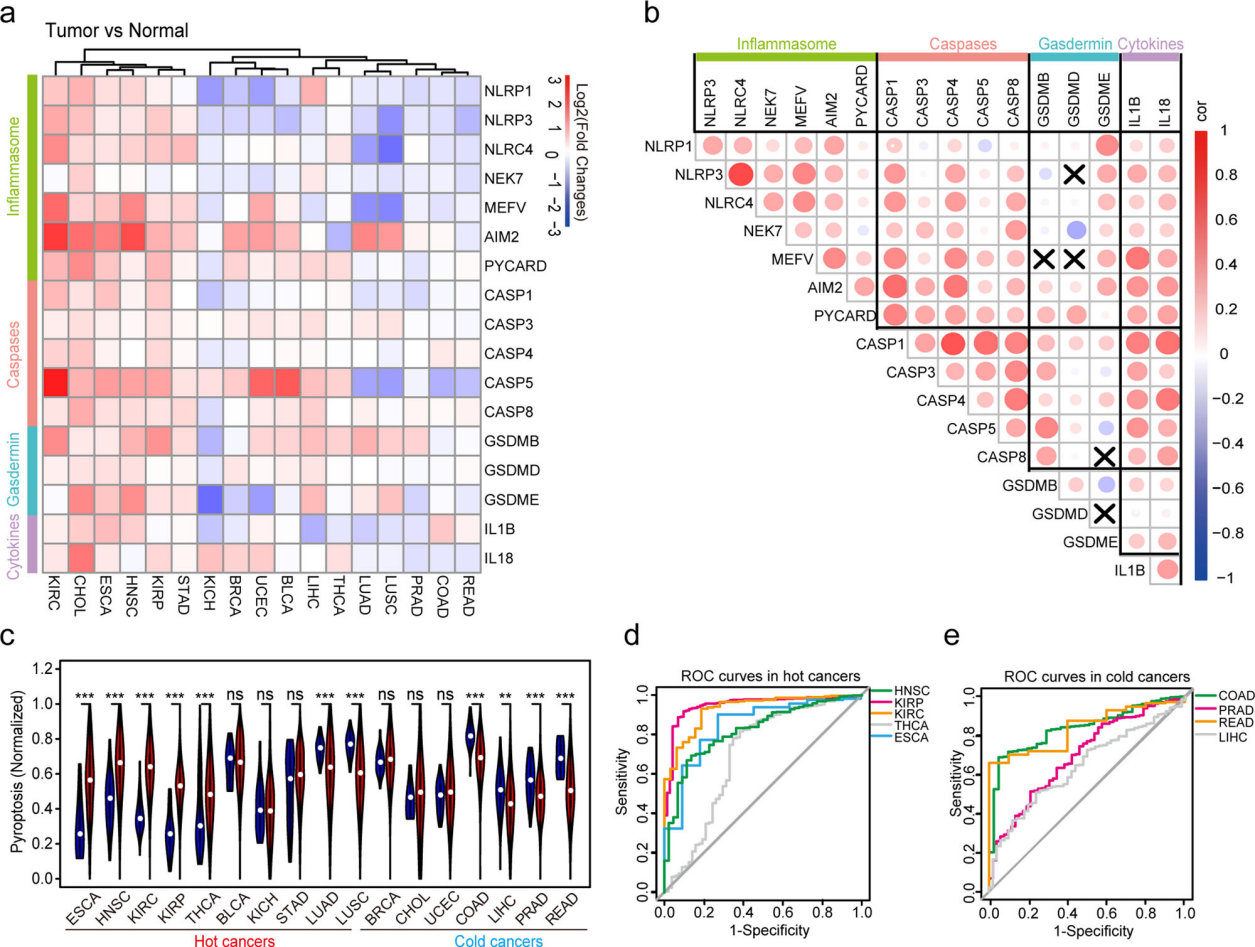
The landscape of pyroptotic factors across 17 cancer types. **a** Heatmap of RNA expression alterations of the 17 pyroptosis-associated genes (PAGs) in 17 cancer types. RNA expression levels were calculated as log2 (average expression of the tumor to normal); **b** The correlation heatmap of the 17 PAGs in the 17 cancer types. X, *P* > 0.05; **c** The violin plots of the normalized enrichment score of the pyroptosis-associated genes (NESPAGs) for normal and tumor samples. The violin filling in red represents tumor samples, while that in blue represents normal tissue. **d**, **e** The receiver operating characteristic curve (ROC) curves for the prediction of normal and tumor samples.

We first determined the RNA expression profiles of the PAGs in 17 types of cancer using data obtained from The Cancer Genome Atlas (TCGA). The four sets of PAGs displayed a similar pattern and dynamic expressions across 17 cancer types (Fig. 1a, Supplementary Table 1). Positive correlations were found between individual genes internally and externally in the four sets of PAGs (Fig. 1b). Therefore, we generated the NESPAGs for the whole pyroptosis assay during tumors development. Cancers were also grouped into “hot cancer” and “cold cancer” to define the high-immune-activity and low-immune-activity cancers, based on the PD-L1 expression and the proportion of TILs (Supplementary Table 2). Interestingly, elevated NESPAGs were found in 5 of the 10 “hot cancers”; whereas reduced NESPAGs were found in 4 “cold cancers” but only in 2 “hot cancers”, when compared with their controls (Fig. 1c). Our observations indicated that pyroptosis may be correlated with tumor immunocompetence.

To explore the diagnostic value of NESPAGs in both “hot” and “cold” cancers, we performed the receiver operating characteristic (ROC) curve analysis. Figure 1d, e showed that NESPAGs could effectively be used to distinguish between tumor and normal tissues in both groups. In “hot cancers”, the area under the curve (AUC) value of NESPAGs for head and neck squamous carcinoma (HNSC) was 0.821 (95% CI 0.763–0.880), for kidney renal papillary cell carcinoma (KIRP) was 0.929 (95% CI 0.887–0.971), for kidney renal clear cell carcinoma (KIRC) was 0.948 (95% CI 0.922–0.974), for thyroid carcinoma (THCA) was 0.698 (95% CI 0.611–0.785), and for esophageal carcinoma (ESCA) was 0.846 (95% CI 0.722–0.969). On the other hand, the AUC of NESPAGs for colon adenocarcinoma (COAD) was 0.836 (95% CI 0.785–0.887), for liver hepatocellular carcinoma (LIHC) was 0.645 (95% CI 0.572–0.718), for liver hepatocellular carcinoma (READ) was 0.835 (95% CI 0.749–0.922), and for prostate adenocarcinoma (PRAD) was 0.695 (95% CI 0.622–0.768) in “cold cancer”. We also investigated the genetic variations of the PAGs in 33 cancer types. However, a very low frequency of somatic mutations (median = 0.002), copy number amplifications (median = 0.018), and copy number deletions (median = 0.017) was found for an individual of 17 PAGs (Supplementary Tables 3–5 and Supplementary Figs 1, 2). Taken together, these results indicated that PAGs are associated with tumor diagnosis that could discriminate between tumor and normal samples, based on their tumor immune activity during cancer development.

### Pyroptosis correlated with immune infiltration and immune activation

**Fig. 2:**
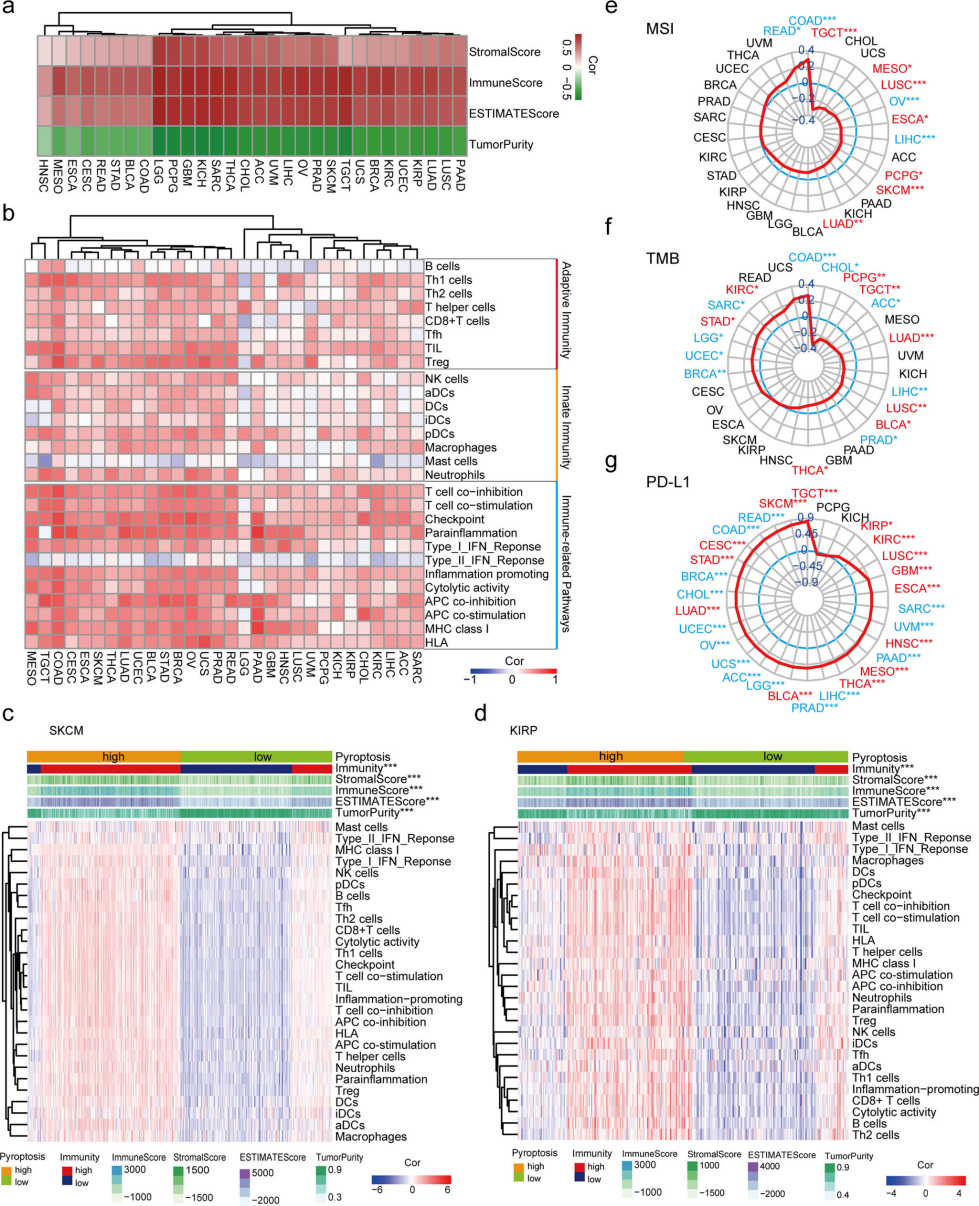
Correlation analysis between pyroptosis signature and immune-related signatures across 30 cancer types. **a**, **b** The relationship between normalized enrichment score of the pyroptosis-associated genes (NESPAGs) and tumor environment scores (**a**), as well as immune-related signatures (**b**). **c**, **d** The heatmap of tumor microenvironment scores and immune activity between high and low NESPAGs group in skin cutaneous melanoma (SKCM) (**c**) and kidney renal papillary cell carcinoma (KIRP) (**d**) cohorts. **e**–**g** The correlation of pyroptosis with microsatellite instability (MSI) (**e**), tumor mutational burden (TMB) (**f**), and programmed death-ligand 1 (PD-L1) (**g**) across 30 cancer types. *P* values were calculated using the Spearman correlation test or Wilcoxon rank-sum test (*P < 0.05; **P < 0.01; ***P < 0.001). The blue color text indicates “cold cancer”, while the red color text indicates “hot cancer”. Tfh T follicular helper, TIL tumor-infiltrating lymphocyte, aDC activated dendritic cells, iDC immature DCs, NK cells natural killer cells, pDC plasmacytoid dendritic cells.

To further understand the interaction between pyroptosis and the tumor microenvironment, we first calculated the proportion of immune cells, stromal cells, and tumor purity for tumor environment assessment^29^. Intriguingly, we found that NESPAGs showed global strong positive correlations with immune cells or stromal cells, while they showed negative correlations with tumor purity in each cancer (Fig. 2a). Furthermore, we also quantified the proportions of immune-cell types in 30 types of cancers using a deconvolution algorithm of CIBERSORT^30^. Among the 22 immune cells that had infiltrated the tumor microenvironment, the CD8+ T cells showed a positive correlation with NESPAGs in a majority of cancers with statistical significance (9/10) (Supplementary Fig. 3a).

The relationship between pyroptosis and immune activity was further assessed using 28 immune-related signatures, which included adaptive and innate immunity, as well as the common immune-related pathways. Single sample gene set enrichment analysis showed that most immune-related signatures were strongly and positively correlated with NESPAGs across the 30 cancer types (Fig. 2b). Additionally, we applied hierarchical clustering to quantify and qualify immune activity in both SKCM and KIRP cohorts (Supplementary Fig. 3b, c). We found a high level of NESPAGs appearing an elevated immune activity, immune scores, and stromal scores, and an inverse trend of tumor purity in both SKCM and KIRP cohorts (Fig. 2c, d). These observations suggested that elevated expression of NESPAGs was positively correlated with the proportion and activity of immune cells in the tumor microenvironment, especially in high-immune-activity cancers.

Since the MSI or TMB presence and PD-L1 expression were associated with the outcome of cancer patients treated with immune checkpoint inhibitors^31–33^, we next analyzed the correlation of pyroptosis with MSI, TMB, and PD-L1. As shown on the radar maps, we found that NESPAGs were negatively related to MSI in a majority (77.8%) of “hot cancers”. Only 2 “cold cancers” showed a positive correlation, in which COAD showed the strongest positive correlation (Fig. 2e. coefficient = 0.293, *p* < 0.001). In addition, NESPAGs expression was positively correlated with TMB in 5 out of the 7 “cold cancers” (Fig. 2f). Since the “hot cancer” and “cold cancer” were grouped according to immunotherapy sensitivity, we indeed found that the positive correlation between pyroptosis and PD-L1 in “cold cancer” was much stronger than that of “hot cancer” (Fig. 2g). Thus, similar to PD-L1, the induced-pyroptosis expression might improve the susceptibility to tumor immunotherapy.

### Pyroptosis altered immune activity in cancer cells

**Fig. 3:**
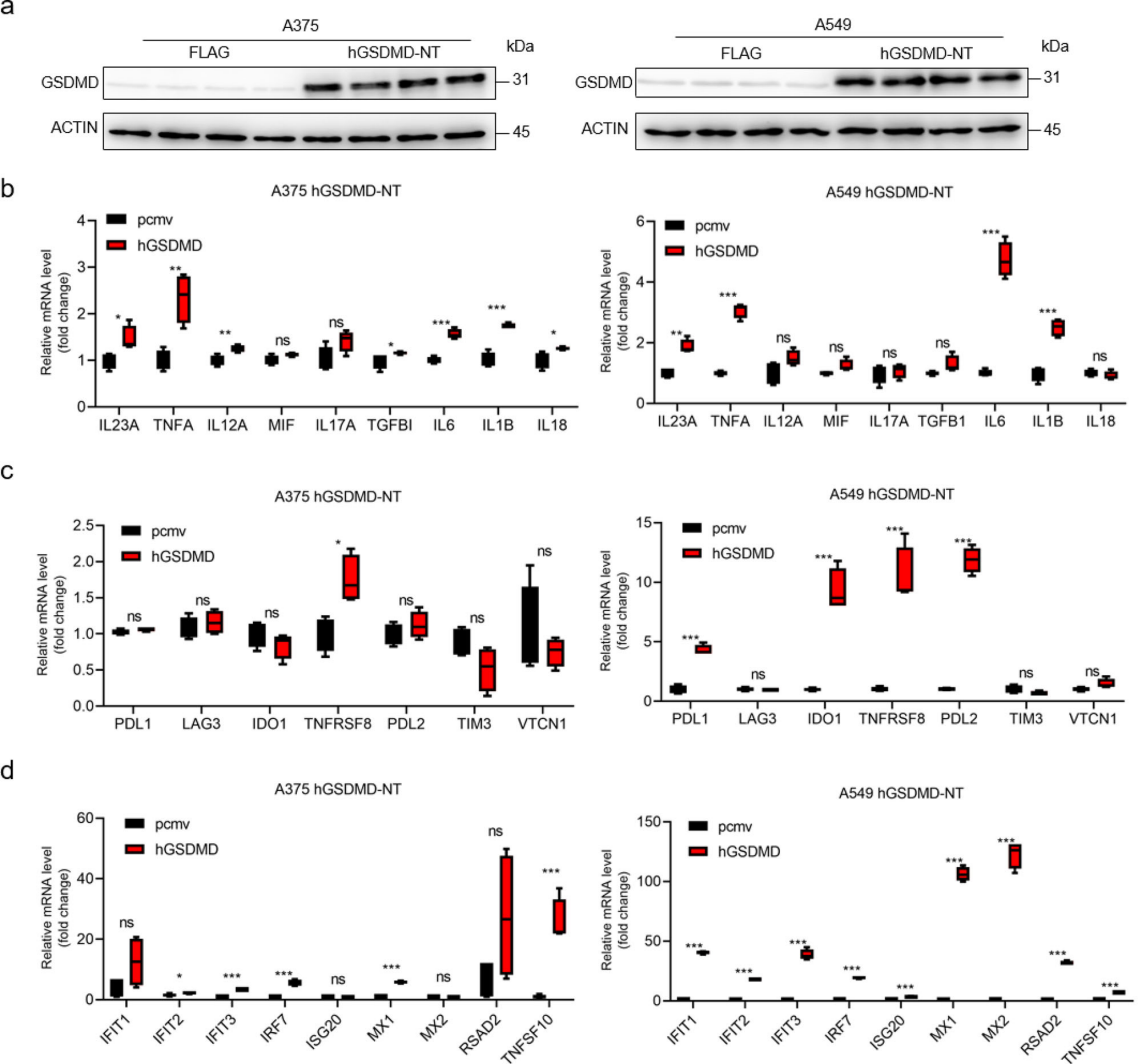
Pyroptosis altered immune activity in cancer cells. **a** Western blot assay showing the successfully upregulated GSDMD-NT by GSDMD-NT plasmid transfection. **b** qPCR analysis of inflammatory cytokines gene expression in A375 and A549 cell lines with GSDMD-NT plasmid transfection or its control. GAPDH was used as an internal control for qPCR analysis. **c** qPCR analysis of immune checkpoint-related genes expression in A375 and A549 cell lines with control or GSDMD-NT plasmid. **d** qPCR analysis of the Type I TNF response pathway-related genes expression in A375 and A549 cell lines with control or GSDMD-NT plasmid. Data was mean ± Standard Deviation (SD). *P* values were calculated using the Spearman correlation test or Student’s t-tests after the assessment of normality (*n* = 4/group, *P < 0.05; **P < 0.01; ***P < 0.001).

To confirm the correlation between pyroptosis and immune, we stimulated pyroptosis by overexpressing GSDMD-NT in A375 and A549 cells^9^. Western blot assay showed that GSDMD-NT was successfully upregulated by transfection of GSDMD-NT plasmid (Fig. 3a and Supplementary Fig. 4). The inflammatory cytokines (*IL23A*, *TNFα*, *IL12A*, *MIF*, *IL17A*, *TGFB1*, *IL6*, *IL1B* and *IL18*) and immune checkpoint-related genes (*PD-L1, LAG3*, *IDO1*, *TNFRSF8*, *PDL2*, *TIM3*, and *VTCN1*) were significantly elevated in GSDMD-NT overexpressed A549 and A375 cell lines by qPCR assay (Fig. 3b, c). Interestingly, the gene expressions of the type I TNF response pathway (*IFIT1*, *IFIT2*, *IFIT3*, *IRF7*, *ISG20*, *MX1*, *MX2*, *RSAD2*, and *TNFSF10*) were increased by transfection of GSDMD-NT plasmid in both A375 and A549 cell lines (Fig. 3d). Consistent with bioinformatical analysis, our experimental data suggested that pyroptosis altered tumor immunity.

### The prognostic value of pyroptosis in skin cutaneous melanoma

**Fig. 4:**
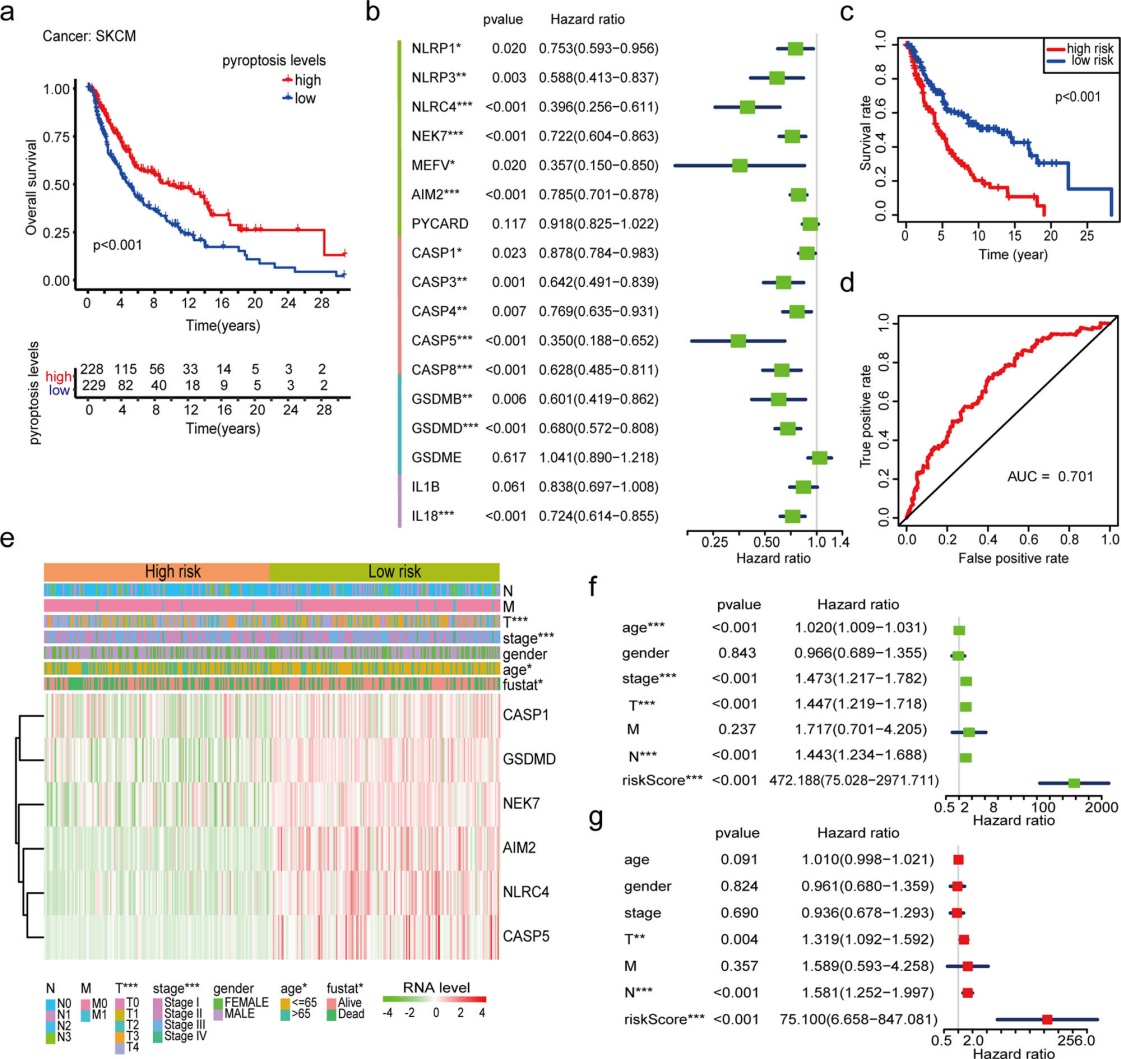
The prognostic value of the pyroptosis-associated genes (PAGs) in the skin cutaneous melanoma (SKCM) cohort. **a** The Kaplan–Meier survival plots of patients grouped by the global expression pattern of PAGs in the SKCM cohort. **b** The distribution of the hazards ratios of the 17 PAGs. **c** The pyroptosis score model (PSM) for the prediction of the survival of SKCM patients. **d** The survival plots of patients were grouped based on the global expression pattern of the risk score in the SKCM cohort. **e** The heatmap of pyroptotic factor expression and clinical characteristics in the SKCM cohort. **f**, **g** Univariate (**f**) and Multivariate (**g**) cox regression analysis of PSM and clinical characteristics of the SKCM cohort. *P* values were calculated using Fisher’s exact test or Cox test (*P < 0.05; **P < 0.01; ***P < 0.001).

Since tumor-infiltrating immune activity could affect and predict the prognosis of cancer patients^34^. Next, we explored the prognostic value of the PAGs in 33 cancer types using a cox proportional hazards regression analysis (Supplementary Fig. 5). Interestingly, NESPAGs served as an indicator of poor prognosis in most “cold cancers”, such as LAML, brain lower-grade glioma (LGG), pancreatic adenocarcinoma (PAAD), and uveal melanoma (UVM). Conversely, an opposite result was found for “hot cancers” of the SKCM cohort (HR = 0.236; 95% CI = 0.124–0.450; *p* < 0.001). Similarly, the Kaplan–Meier survival plot confirmed that a high level of NESPAGs indicated a longer survival time than that of a low level of NESPAGs in patients with SKCM (Fig. 4a).

Next, we determined whether the individual pyroptotic factors that could be used to predict SKCM prognosis using a cox proportional hazards regression analysis. As expected, up to 14 of 17 PAGs were considered as biomarkers of SKCM prognosis (Fig. 4b). To improve model accuracy and decrease model overfitting, we carried out a LASSO analysis and established a risk model based on the expression of 6 PAGs (*CASP5*, *NEK7*, *AIM2*, *CASP1*, *NLRC4*, and *GSDMD*) (Supplementary Fig. 6a, b). The model was further divided into a high-risk and low-risk group based on the median risk score (0.159). Interestingly, the low-risk group presented a survival benefit, compared with the high-risk group, as shown through survival curves (Fig. 4c). The risk model displayed a good performance in predicting the 5-year survival rates of the SKCM cohort using the ROC curve analysis (AUC = 0.701, Fig. 4d). In addition, we found that the risk model was closely correlated with the clinical features of the SKCM cohort, including age, stage status, and tumor size (Fig. 4e, Supplementary Table 6). The univariate Cox regression analysis showed that age, tumor stage, tumor size, lymphatic metastasis, and risk score were associated with poorer survival of SKCM patients. Moreover, the risk score predicted a worse outcome independent of tumor size, lymphatic metastasis, age, and tumor stage, which was analyzed using multivariate Cox regression adjusted by tumor purity (Fig. 4f, g). These results revealed that pyroptotic factors played a vital role in the prognosis and progression of SKCM.

The PAGs role in SKCM molecular classification was further determined. We classified SKCM patients into three groups using the consensus clustering approach based on the expression profiles of the 17 PAGs (Supplementary Fig. 7a, b). Interestingly, we found that SKCM patients in the cluster1 subgroup showed better overall survival than those in the other two subgroups (Supplementary Fig. 7c). Moreover, we found that clinical characteristics of tumor size and stage status were significantly correlated with three cluster types (*p* < 0.05, Supplementary Table 7, Supplementary Fig. 7d). These results suggested that the elevated level of pyroptosis indicated a better survival and was strongly correlated with clinicopathological features, which could probably be used for the molecular classification of tumors.

### The predictive role of pyroptosis in response to anti-PD-1 immunotherapy in SKCM

**Fig. 5:**
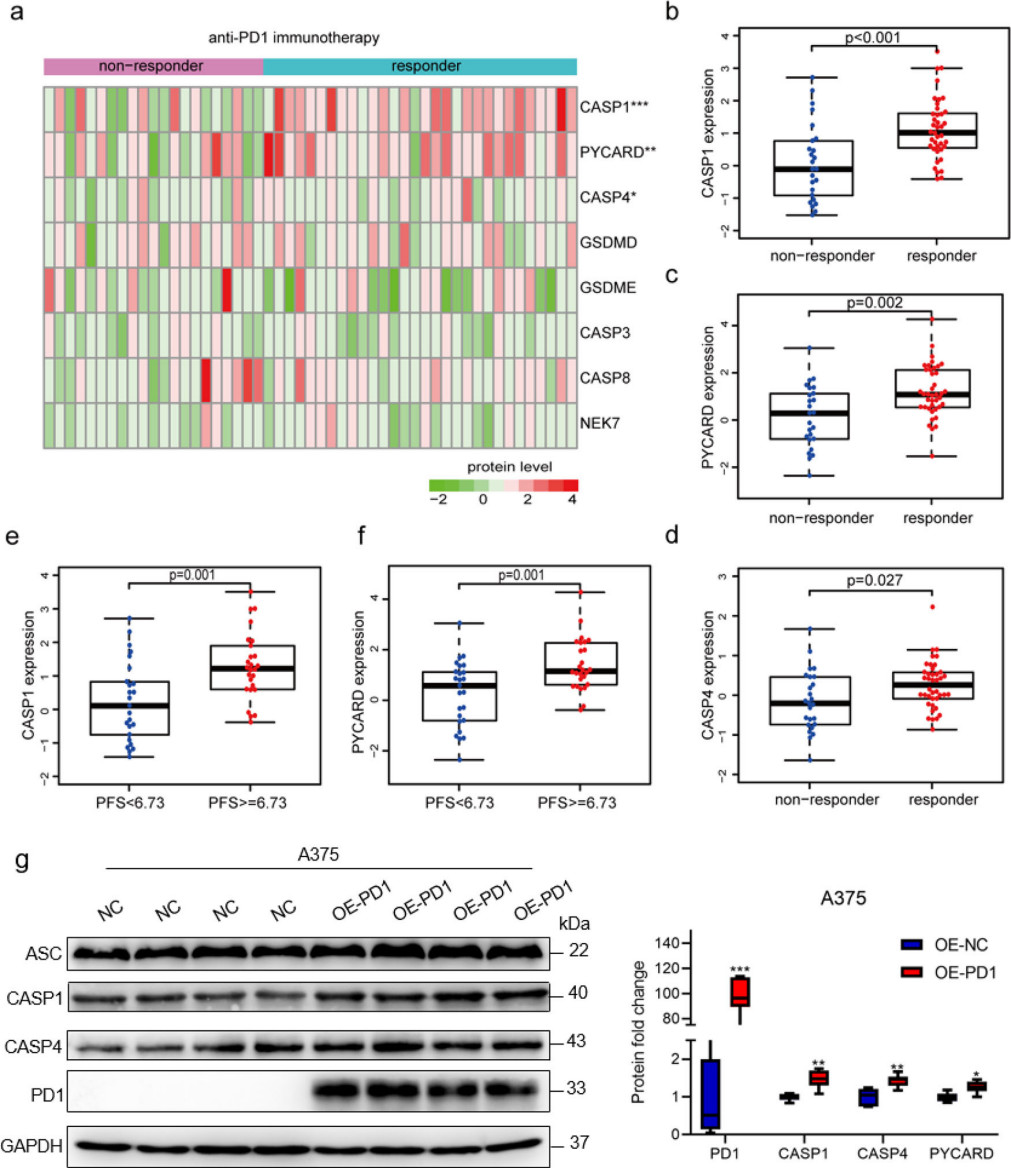
The fold-changes of the pyroptosis-associated protein in the anti-PD-1 immunotherapy cohort. **a** The heatmap of pyroptotic factor protein expression between non-responders and responders in the anti-PD-1 immunotherapy cohort. **b**–**d** The protein expression levels of pyroptotic factors CASP1 (**b**), PYCARD (**c**), and CASP4 (**d**) between responders and non-responders. **e**, **f** The protein expression levels of pyroptotic factors CASP1 (**e**) and PYCARD (**f**) in patients with a disease progression-free survival value of <6.73 and >=6.73. The median duration of disease progression-free survival was 6.73 months. **g** Western blot analysis for protein expression in A375 cells after transfection of PD1. GAPDH was used as a normalized control. Data was mean ± Standard Deviation (SD). *P* values were calculated using the Student’s t-tests after the assessment of normality (*P < 0.05; **P < 0.01; ***P < 0.001. *n* = 7/group).

Recently, immune-checkpoint inhibitors, such as PD-1/PD-L1 antibodies, have significantly improved the survival of partial patients with melanoma^35^. To further identify the predictive role of pyroptosis in immunotherapy, we determined the protein levels of PAGs in 51 melanoma patients treated with anti-PD-1 therapy from the study conducted by Harel et al.^36^. Based on the impact of the treatment, patients were categorized as responders (*n* = 30) and non-responders (*n* = 21). The responders showed a better prognosis, compared with non-responders (Supplementary Fig. 8). Then, we accessed the proteomic data of melanoma response to immunotherapy to explore potential biomarkers of pyroptosis^36^ (Fig. 5a). Not surprisingly, we found that the protein levels of CASP1, PYCARD, and CASP4 in the responder group were significantly higher than that of the non-responder group (Fig. 5b–d). Interestingly, higher protein level expression of CASP1 and PYCARD indicated better progression-free survival (Fig. 5e, f). Evidence reported that *PD-1* overexpression increased the efficacy of anti-PD-1 therapy for human melanoma^37^. Therefore, we overexpressed *PD-1* in the A375 cell line to establish PD-1 immunotherapy-sensitive melanoma cell lines. Consistently, CASP1, CASP4 and PYCARD, were increased in overexpressed *PD-1* of A375 cells by western blot assay (Fig. 5g and Supplementary Fig. 9). The above data proved that CASP1, CASP4 and PYCARD might act as potential biomarkers to predict the effectiveness of anti-PD-1 immunotherapy for melanoma patients. However, more in-depth clinical studies are needed to verify in the future.

## Discussion

This study investigated the expression levels and genetic alterations of 17 PAGs and explored their correlations between pyroptosis and anti-tumor immunity across 30 cancer types. Our results indicated that elevated pyroptosis was shown in cancers with high-immune activity, but reduced in low-immune activity tumor patients. Pyroptosis could distinguish between tumor and normal tissues. Pyroptosis was also positively correlated with immune infiltration and biomarkers of ICB therapy, such as TMB, MSI, and PD-L1 expression. In SKCM, the risk model was established on the PAGs expression profile and showed a strong performance in predicting their prognosis. Moreover, the protein levels of pyroptotic factors could act as potential prognostic biomarkers for cancer immunotherapy. In conclusion, we determined the pan-cancer effect of PAGs and provide broad insights into the clinical application of pyroptosis for anti-tumor immunotherapy.

At present, the specific mechanism of pyroptosis in tumors is still controversial. Hence, we comprehensively analyzed the expression and gene variation of pyroptosis-related factors in 33 types of cancer, which provided a theoretical basis for exploring the mechanism of pyroptosis in tumors. Importantly, the immune status of the tumors, indicated as “hot” or “cold” cancers, was significantly related to the expression levels of the PAGs, which also complemented the current mechanism of pyroptosis and immunity research^26–28^. Moreover, as fully described in this study, we fortunately found that the AUC of NESPAGs showed a great correlation with tumor diagnosis in some types of “hot cancers”. However, many factors cause the occurrence or enhancement of pyroptosis, such as pathogen infection or inflammation, etc. Interestingly, the composition of the microbiome varies with tumor type, which is related to the clinical outcome of patients treated with immune checkpoint inhibitors^38^. These results suggest that pyroptosis may be at least partly responsible for anti-tumor immunity.

Moreover, the gasdermin family includes GSDMA, GSDMB, GSDMC, GSDMD, and GSDME. GSDMB and GSDME, which have been reported to be associated with anti-tumor immunity in human cancer^26,28,27^. In addition, meta-analysis has shown that the inflammatory response activated by gasdermin could enhance the favorable outcome of ICB therapy by increasing the number of infiltrated lymphocytes^39^. In our study, “hot cancers” such as KIRC, ESCA, HNSC and KIRP, indicated high expression levels of the gasdermin family members in tumors, compared with normal tissue. On the contrary, PRAD, COAD, and READ of “cold cancers” showed opposite expression trends. Our findings pointed out the correlation between pyroptosis and anti-tumor immunity based on bioinformatical analysis and experiments in vitro. Overall, pyroptosis is a promising target in tumor immunotherapy, and more experiments in the animal model and clinical trials are needed to be carried out. There is a long way to go before the clinical application of pyroptosis for anti-tumor immunotherapy. These findings may guide the development of a novel class of therapeutics for the treatment of low immunocompetence patients.

Melanoma is one of the most common malignancies with a high level of metastasis in clinical settings and patients with an advanced stage are relatively sensitive to immunotherapy^40^. Our results indicated that the majority of the PAGs significantly affected the prognosis of patients with melanoma and are strongly associated with the clinical stage and tumor size. Therefore, the induction of pyroptosis in tumor cells may be used as a potential alternative therapy^26^. Emran et al. reported that low levels of expression of inflammasome components prompted the poor prognosis of melanoma^41^. It shows a dual role in melanoma, indicating that it could accelerate melanoma growth by activating inflammasomes and by suppressing apoptosis^42^. The results may be explained by the dominant function of the pyroptosis executor in melanoma. Interestingly, ASC showed a stage-dependent dual role in primary and metastatic melanoma. Compared with primary tumors, the inhibitory effect of ASC in metastatic melanoma was gradually converted into a tumorigenic role through the improvement of NF-κB pathways and the secretion of inflammasome-medicated IL-1β^43^. Therefore, pyroptosis could play a dual role in early and advanced tumors. During the early stage, pyroptosis could provide an appropriate immune microenvironment through its proinflammatory effect. As the tumor progresses, its anti-tumor immunity plays a dominant role in the advanced stage. However, a more systematic and theoretical analysis is urgently required to confirm the mechanism of pyroptosis in tumors.

At present, immune checkpoint inhibitors (such as anti-PD-1/PD-L1) are recognized as the most prominent clinical successes in melanoma. Anti-PD-1 antibodies can suppress tumor immune escape by targeting PD-L1 ligands, activating cytotoxic T cells, and triggering anti-tumor responses^44^. TMB, MSI, and PD-L1 are common clinical biomarkers that can predict the treatment efficacy of PD-1/PD-L1 checkpoint inhibitors^45–47^. A recent pan-cancer analysis showed that GSDM family expression, which are the executors of pyroptosis related to MSI and TMB in some specific types of cancers (BRCA, COAD, READ, GBM, THCA, THYM, and LUSC)^48^. Interestingly, GSDMB, GSDMD, and GSDME were included in the NESPAGs, as a pyroptosis signature established in our study. The NESPAGs had a stronger correlation with immune activation, MSI, and TMB across pan-cancer. Moreover, we also found this correlation was mostly present in “cold cancers” such as BRCA, COAD, and READ. Therefore, NESPAGs could better represent the change of the pyroptosis-related pathway. Activation of the NESPAGs might be a hopeful way to improve the efficacy of immunotherapy. Hence, we speculated that pyroptosis may be involved in predicting the treatment efficacy of immune checkpoint inhibitors. In our bioinformatics and experiment data proved that CASP4 and PYCARD might act as potential biomarkers to predict the effectiveness of anti-PD-1 immunotherapy for melanoma patients. This also provides a method for screening melanoma patients with good immunotherapy efficacy.

In summary, our analysis demonstrated that pyroptosis is involved in tumor development, prognosis and response to ICB therapy through its interaction with tumor immune activity.

## Methods

### Gene list collection

To achieve a comprehensive understanding of pyroptosis, 17 PAGs were divided into four groups that “Inflammasomes” (*NLRP3*, *NLRP1*, *PYCARD*, *NEK7*, *NLRC4*, *MEFV*, and *AIM2*), “Inflammatory Caspases” (*CASP1*, *CASP3*, *CASP4*, *CASP5*, and *CASP8*), “Gasdermin Family” (*GSDMB*, *GSDMD*, and *GSDME*) and “Proinflammatory Cytokines” (*IL-18* and *IL-1β*)^14^.

### Datasets and preprocessing

The genomic, transcriptomic, and clinical data of 33 cancer types were downloaded from the University of California Santa Cruz (UCSC) Xena browser (http://xena.ucsc.edu/) and the case numbers of data were shown in Supplementary Table 8. The RNA-seq data across all tumors and their control samples was normalized as log_2_(FPKM + 1). 17 cancer types were sorted for the differential gene expression between cancer and normal, excluding the normal samples less than 10. We firstly obtained the mean expression values of the individual 17 PAGs in tumor and normal samples respectively, then calculated the differential expression by log_2_ fold-change in each cancer (Supplementary Table 1). The frequency of copy number variation (CNV) and mutation for 17 PAGs was summarized in Supplementary Tables 3–5. We also referenced an article with proteomics data^36^, in which missing values were imputed using the nearest neighbor averaging method in the impute R package^49^.

### Single sample gene set enrichment analysis

To calculate the normalized enrichment score of 28 immune-related signatures in the tumor microenvironment, the single-sample gene set enrichment analysis (ssGSEA) was used and conducted with the GSVA R package (version1.32.0)^50^. Moreover, ssGSEA was also used to calculate the normalized enrichment score of the pyroptosis-associated genes (NESPAGs) of the whole 17 PAGs expression. The spearman analysis was performed between the NESPAGs and normalized enrichment score of 28 immune-related signatures with tumor purity adjusted.

### Identification of differentially NESPAGs

To identify differentially NESPAGs in each cancer type, we used Wilcox’s rank-sum test to identify NESPAGs. The *p* values were adjusted by the FDR method. NESPAGs with adjusted *p* values <0.05 were identified as differentially NESPAGs in each cancer type.

### The correlation of the individual 17 PAGs

The Spearman correlation analysis between 17 PAGs was performed in 17 cancer types, and the results were visualized using the corrplot package (https://github.com/taiyun/corrplot).

### Hierarchical clustering analysis

The hierarchical clustering analysis was used to classify into “hot cancer” and “cold cancer” based on *programmed death-ligand 1* (*PD-L1*) expression and the normalized proportion of tumor-infiltrating lymphocytes (TILs) in 30 types of cancer using the “hclust” function^51,52^. Since the high proportion of immune cells of Acute Myeloid Leukemia (LAML), Lymphoid Neoplasm Diffuse Large B-cell Lymphoma (DLBC), and Thymoma (THYM), these cancers were excluded for clustering analysis. It was also applied to calculate the immune activity in both SKCM and KIRP cohorts according to the NES of 28 immune-related signatures.

### Estimation of immune-cell type fractions

CIBERSORT is a deconvolution algorithm that can be used to characterize the cell composition of complex tissues based on normalized gene expression profiles (GEPs)^53^. In this study, the default set (LM22) was used to quantify the relative proportions of immune-infiltrating cells in 30 cancer types. Normalized gene expression data was analyzed using the CIBERSORT algorithm by running 100 permutations. The spearman analysis was performed between the NESPAGs and immune-infiltrating cells.

### Estimation of the proportion of tumor-infiltrating cells

ESTIMATE is a package used to predict tumor purity, as well as the presence of stromal and immune cells in tumor tissues using gene expression data. ESTIMATE (version1.0.13) was applied to determine the proportion of immune cells and stromal cells in the tumor microenvironment (https://R-Forge.R-project.org/projects/estimate/). Spearman correlation analysis was performed to calculate the coefficient between the estimate scores and NESPAGs.

### Microsatellite instability (MSI) and tumor mutational burden (TMB) estimation in pan-cancer

TMB was defined as the total number of somatic mutations for every one million bases^54^. Here, TMB was calculated for each sample via Perl scripts. MSI was determined based on the number of insertion or deletion events in tumor samples compared with normal ones^55^. Spearman correlation analysis was performed to calculate the coefficient of NESPAGs with TMB, MSI, and PD-L1 for individual cancer, which is shown by radar maps.

### Consensus clustering approach

A consensus clustering approach was performed for the molecular classification of SKCM patients using the ConsensusClusterPlus package^56,57^. In our study, a k value of 3 was considered as the optimal number of groups using the expression signature of the 17 PAGs.

### The pyroptosis score model (PSM) for SKCM

Univariate analysis was first used to determine the prognostic value of the PAGs. The least absolute shrinkage and selection operator (LASSO) regression was performed to identify the most efficient components that contribute to survival prediction using the “glmnet” package. The PSM was established for the prediction of prognosis after the optimal number of feature variables was determined.

### Cell lines and cell culture

The A375 and A549 cell lines were purchased from the National Infrastructure of Cell Line Resources in China (Beijing, China). Cells were cultured in DMEM or RPMI-1640 medium with 10% FBS (Thermo Fisher Scientific, Inc.) and incubated at 37 ˚C with 5% CO_2_.

### Plasmids and transfection

The sequence of *GSDMD-NT* from HEK293 cell line was cloned into the pCMV vector to construct recombinant plasmids named pCMV-GSDMD-NT. The sequence of *PDCD1* from human lymphocyte was cloned into the pCMV vector to construct recombinant plasmids named pCMV-PD1. Cells were transfected with plasmids using Lipofectamine 3000 (Invitrogen) according to the manufacturer’s instructions.

### Western Blot

Western blotting was performed using the standard procedures as we previously reported^58,59^. Proteins were extracted from cells or tissues using RIPA lysis buffer (Solarbio). Equal volumes of lysates were loaded and separated on 10% SDS-PAGE gels and blotted on polyvinylidene difluoride membrane. After being blocked with 5% skim milk for 2 h at room temperature, the membranes were incubated with primary antibodies overnight at 4 ˚C. The primary antibody used in western blot analysis included Anti-ASC (1:1000; ab155970; Abcam); anti-caspase-1 (1:1000; ab207802; Abcam); anti-caspase-4 (1:1000; ab238124; Abcam); anti-GSDMD (1:1000; 66387-1-Ig; Proteintech); anti-PD1 (1:1000; ab52587; Abcam) and anti-GAPDH (1:500; ab8245; Abcam;). Anti-rabbit-HRP (1:5000; #7074, Cell Signaling Technology) and anti-mouse-HRP (1:5000; #7076, Cell Signaling Technology) were used as secondary antibodies and incubated at room temperature for 1 h. The blots were detected using a chemiluminescence kit (cat. no. 34577; Thermo Fisher Scientific, Inc.) and imaged using MiniChemi 610 system (Sage Creation Science, Co., Ltd.).

### RNA extraction and RT-qPCR

Total RNA was extracted from cultured cell lines using TRIzol reagent (Thermo Fisher Scientific, Inc.) according to the manufacturer’s instructions as we previously reported^60,61^. cDNA was synthesized with PrimeScript™ IV 1st strand cDNA Synthesis Mix (Takara, China). qPCR analysis was performed using the Taq Pro Universal SYBR qPCR Master Mix (Vazyme Biotech Co., Ltd.), and *GAPDH* was used as an internal control. Each assay was carried out in triplicates in a Light Cycler 480 Instrument (Roche). The primers for RT-qPCR are shown in Supplementary Table 9.

### Statistics and reproducibility

All experiments were performed in at least biological triplicate with similar results and compared using Student’s t-tests or Wilcoxon tests in the Graphpad Prism software. Statistical analyses were performed using R software (version 3.6.2, www.r-project.org). Continuous variables were expressed as mean ± standard deviation and analyzed using Student’s *t*-tests or Wilcoxon tests. Categorical variables were compared by the Chi-square test or Fisher’s exact test. Correlation analysis used the Spearman method, which was performed using the “cor.test” function. Survival analysis was performed using the Kaplan–Meier method and compared through log-rank tests using the “survival” and “survminer” packages. Multivariate Cox regression models were trained using the “coxph” function of the “survival” package. All tests were two-sided and *p* < 0.05, unless specifically stated, was considered to indicate statistical significance.

### Reporting summary

Further information on research design is available in the Nature Research Reporting Summary linked to this article.

## Supplementary information

Supplementary Information

Description of Additional Supplementary Files

Supplementary Data 1

Reporting Summary

## Data Availability

The genomic, transcriptomic, and clinical data of 33 cancer types were downloaded from the UCSC Xena browser (http://xena.ucsc.edu/). Source data for graphs is available in Supplementary Data 1. Uncropped blot/gel images are available as Supplementary Fig. 4 and 9. Software and resources used for the analyses are described in each method section. Plasmids for pCMV-GSDMD-NT and pCMV-PD1 expression are deposited at Addgene (Accession numbers 188239 and 188240).
