# Supplementary material for: Pyroptosis correlates with tumor immunity and prognosis
Source: Commun Biol. 2022 Sep 6;5:917. doi: 10.1038/s42003-022-03806-x (PMC9448722; doi:10.1038/s42003-022-03806-x)
Supplement: Supplementary file 1 — Supplementary Information [file 42003_2022_3806_MOESM1_ESM.pdf]

Supplementary Information

Pyroptosis correlates with tumor immunity and prognosis

Xiaoying Lou[1†], Kexin Li[1†], Benheng Qian[2], Yiling Li[1], Donghong Zhang[3*] and Wei Cui[4*]

[1]State Key Laboratory of Molecular Oncology, Department of Clinical Laboratory, National Cancer Center/National Clinical Research Center for Cancer/Cancer Hospital, Chinese Academy of Medical Sciences and Peking Union Medical College, Beijing, 100021, China;

[2]Department of Cardiology, The Second Affiliated Hospital of Wenzhou Medical University, 109 Xueyuan Road, Wenzhou 325027, Zhejiang, PR China;

[3]Center for Molecular and Translational Medicine, Research Science Center, Georgia State University, Atlanta, GA 30303, USA;

[4]Department of Clinical Laboratory, National Cancer Center/National Clinical Research Center for Cancer/Cancer Hospital, Chinese Academy of Medical Sciences and Peking Union Medical College, Key Practice of Laboratory Medicine in Qinghai Province, Beijing, 100021, China;

†contributed equally to this work

*Corresponding author

Wei Cui

Department of Clinical Laboratory, National Cancer Center/National Clinical Research Center for Cancer/Cancer Hospital, Chinese Academy of Medical Sciences and Peking Union Medical College, Key Practice of Laboratory Medicine in Qinghai Province, Beijing, 100021, China

Tel: (86)13522649418

Email: cui123@cicams.ac.cn.

Supplementary information contains 9 figures and 9 table.

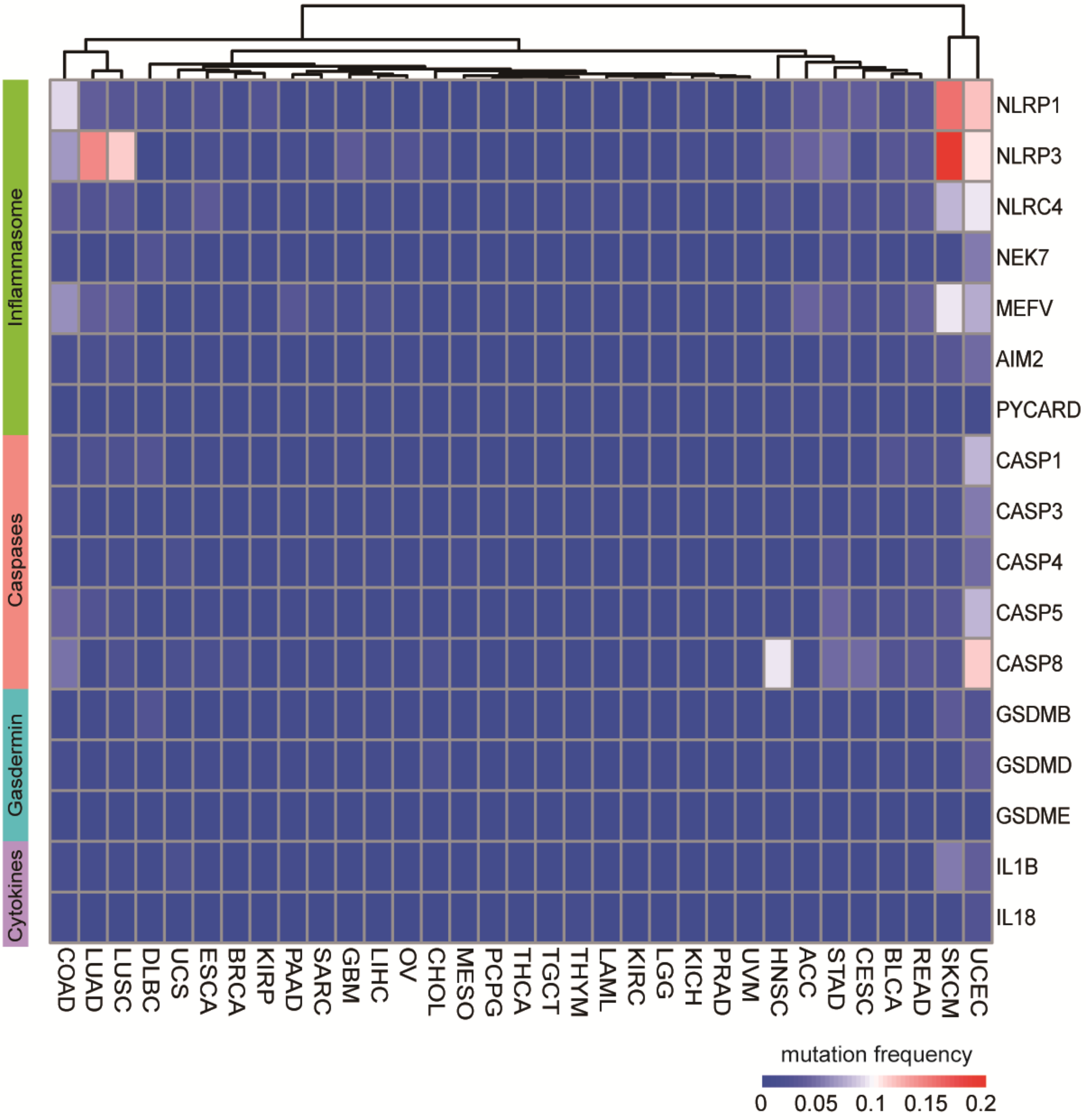

Supplementary Figure 1**.** Mutation frequency of the pyroptosis-associated genes (PAGs) across 33 cancer types.

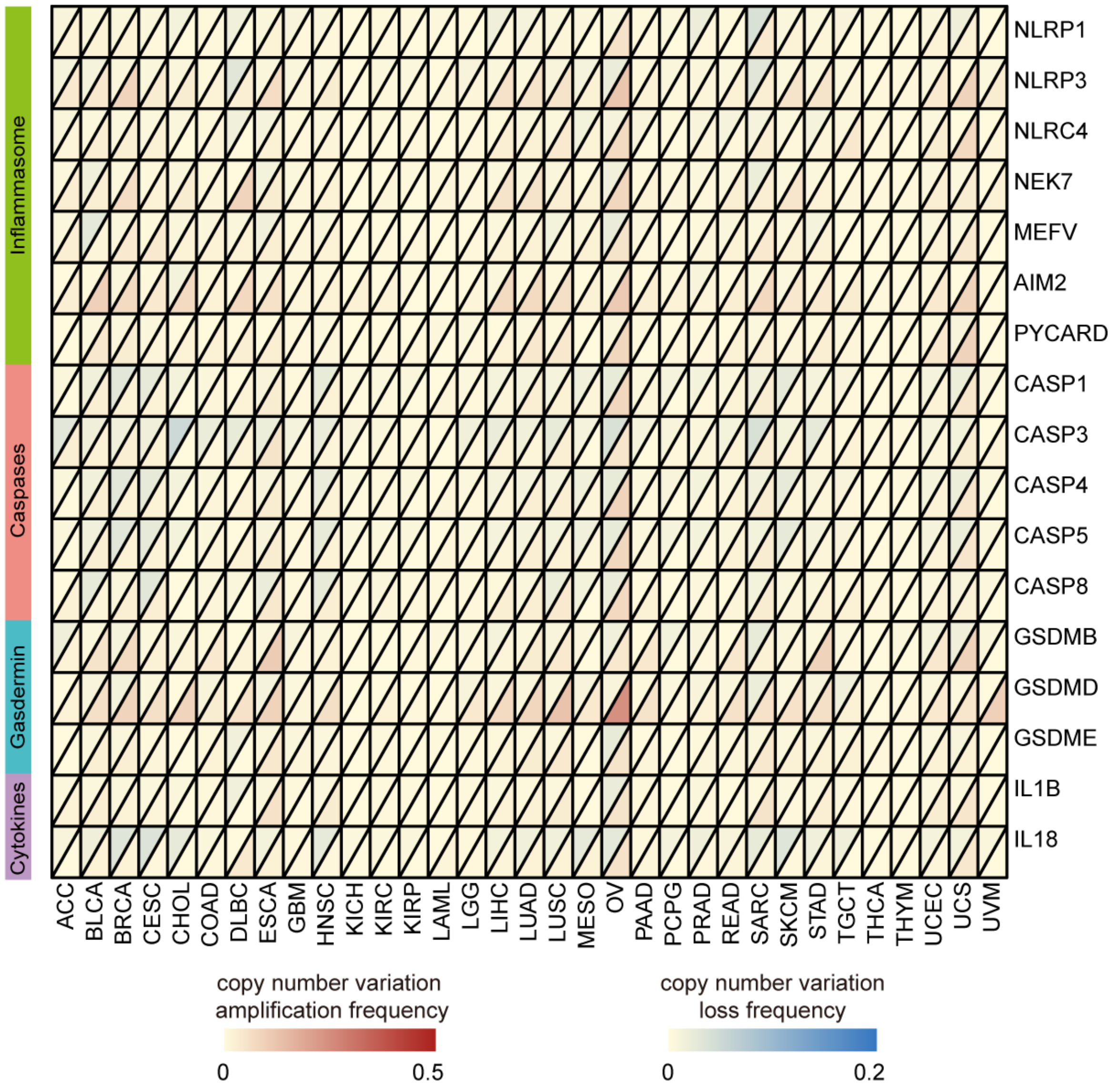

Supplementary Figure 2**.** The frequency of copy number variation for pyroptosis-associated genes (PAGs) across 33 cancer types. The left upper section of each grid shows the deletion frequency, and the right lower section shows the amplification frequency.

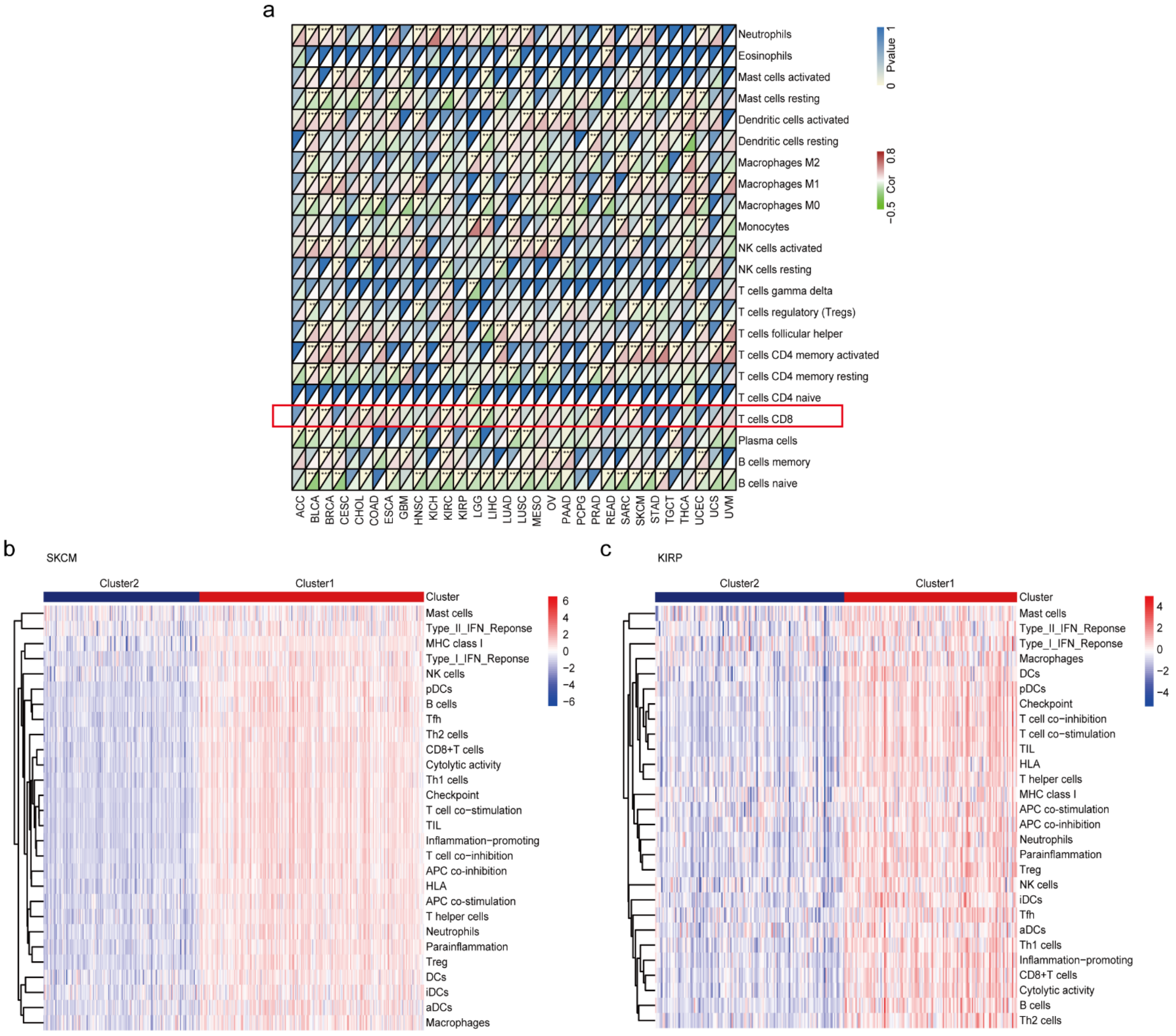

Supplementary Figure 3**.** Survival analysis based on the pyroptosis-associated genes (PAGs) across different cancer types.(a)**.** The correlation between immune-infiltrating cells and normalized enrichment score of the pyroptosis-associated genes (NESPAGs) across 30 cancer types. (b and c)**.** The clustering of 28 immune signatures in the skin cutaneous melanoma (SKCM) cohort (b) and kidney renal papillary cell carcinoma (KIRP) cohort (c).

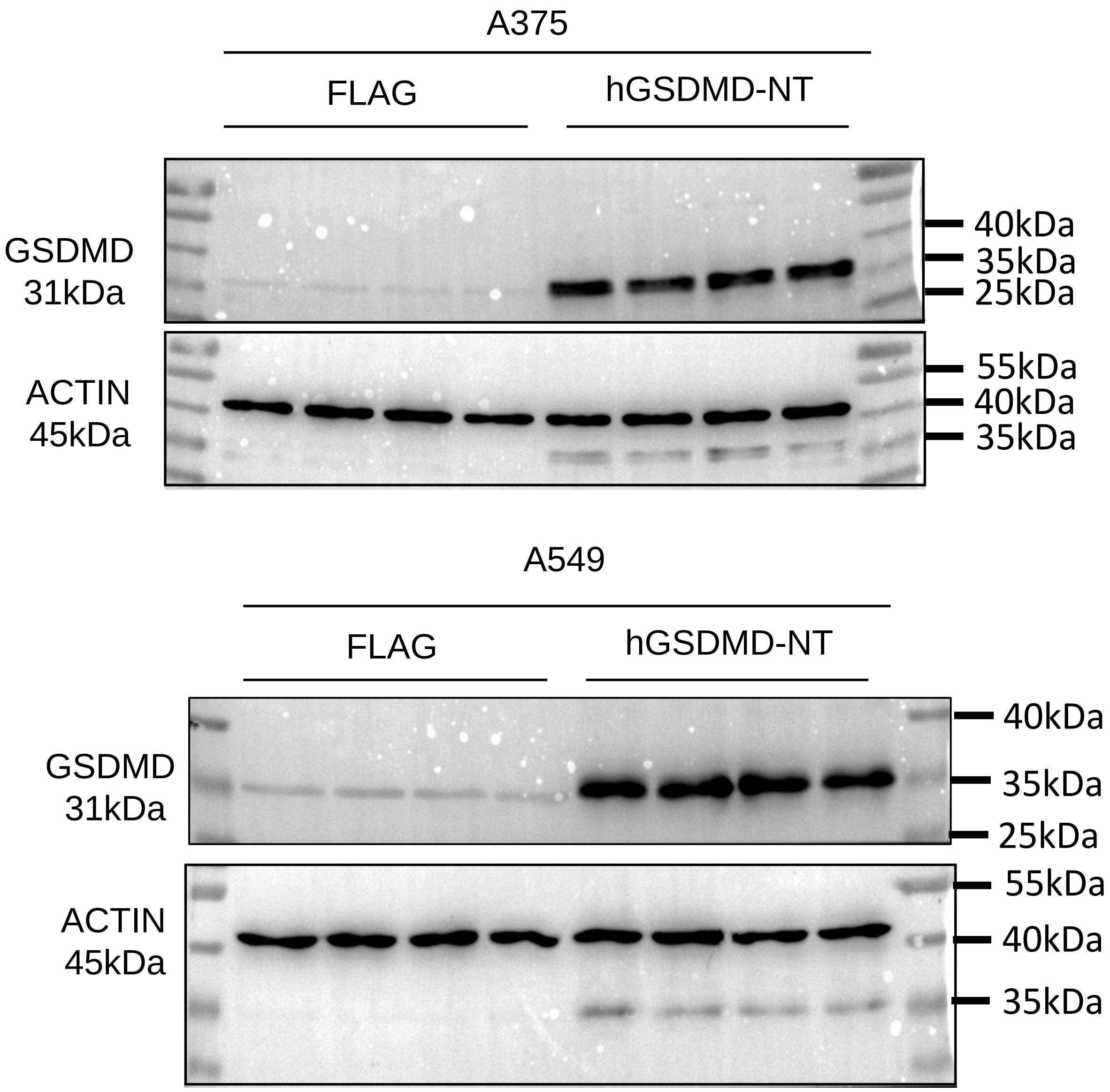

Supplementary Figure 4. Unedited gel for Figure 3a

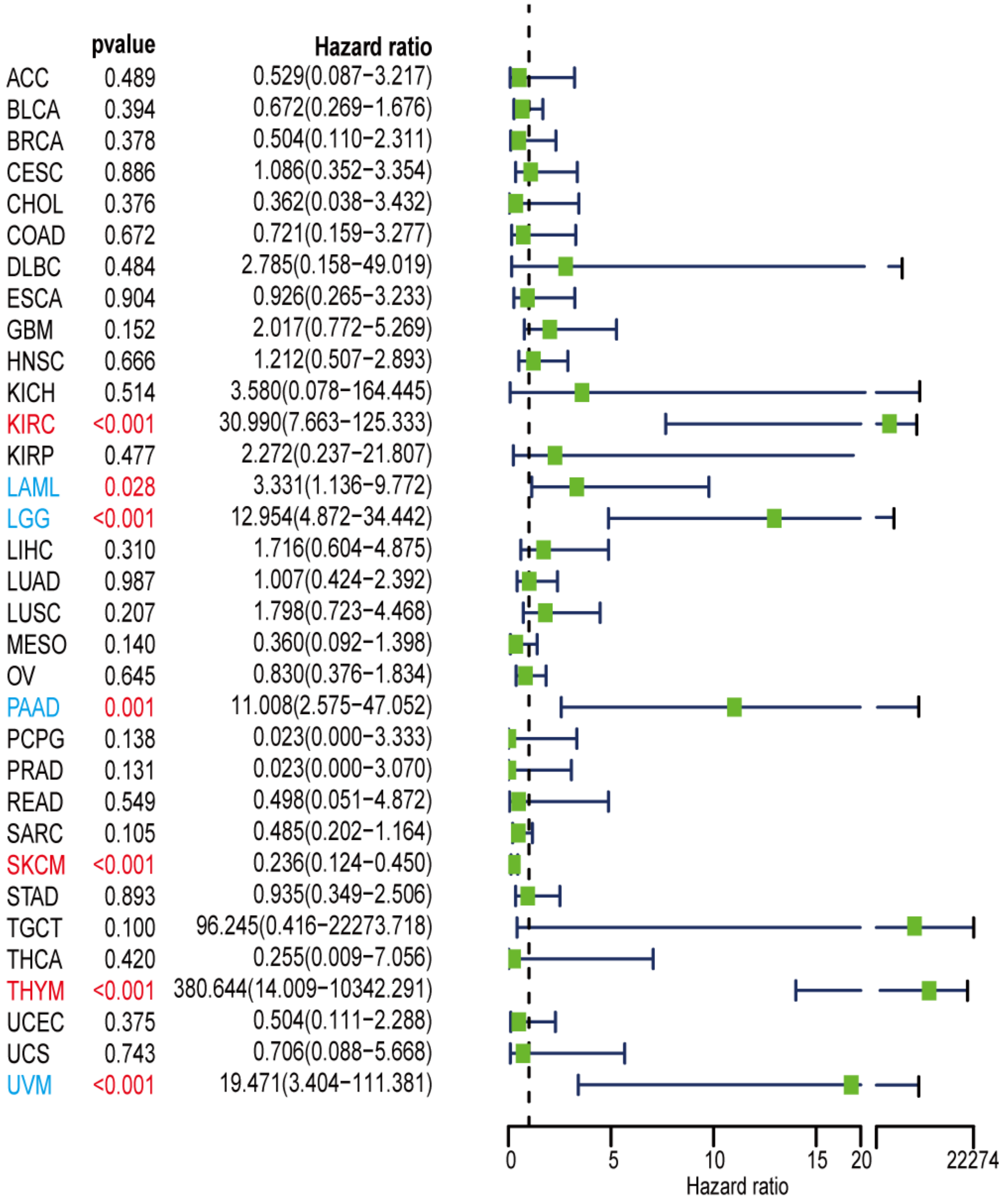

Supplementary Figure 5. The hazards ratios of normalized enrichment score of the pyroptosis-associated genes across 33 cancer types.

a

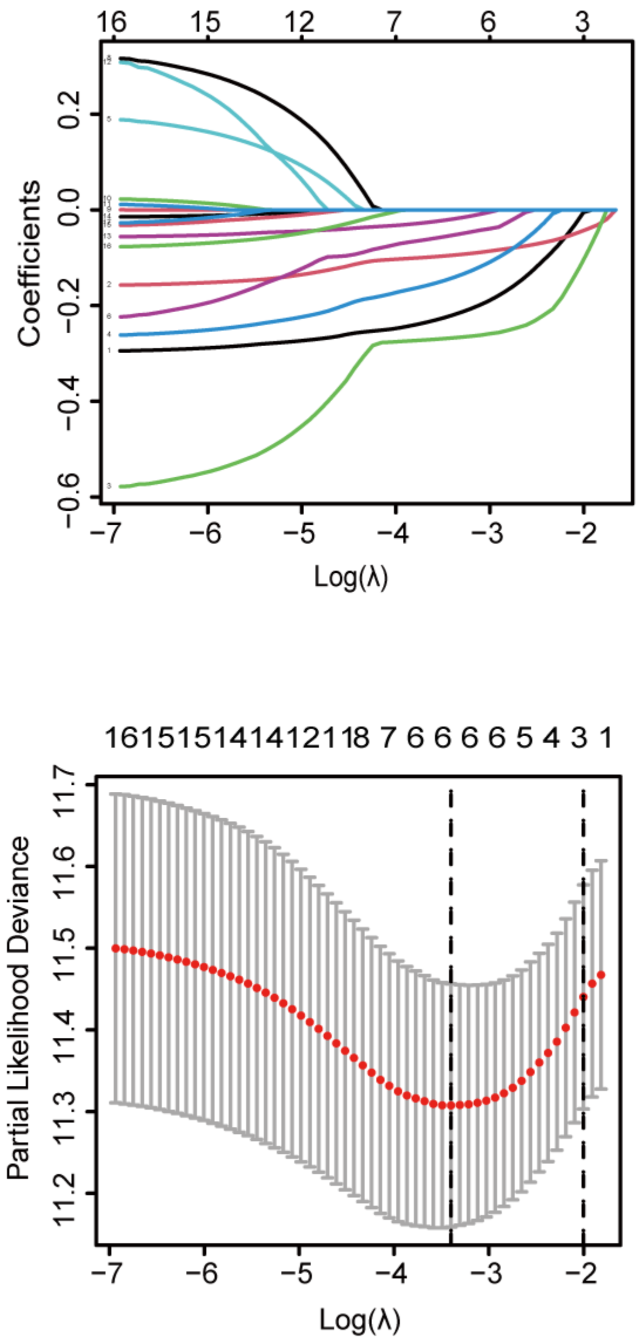

Supplementary Figure 6. Least absolute shrinkage and selection operator regression of the 14 pyroptotic factors in SKCM cohort; (a and b). LASSO analysis to determine the optimal number of characteristic variables.

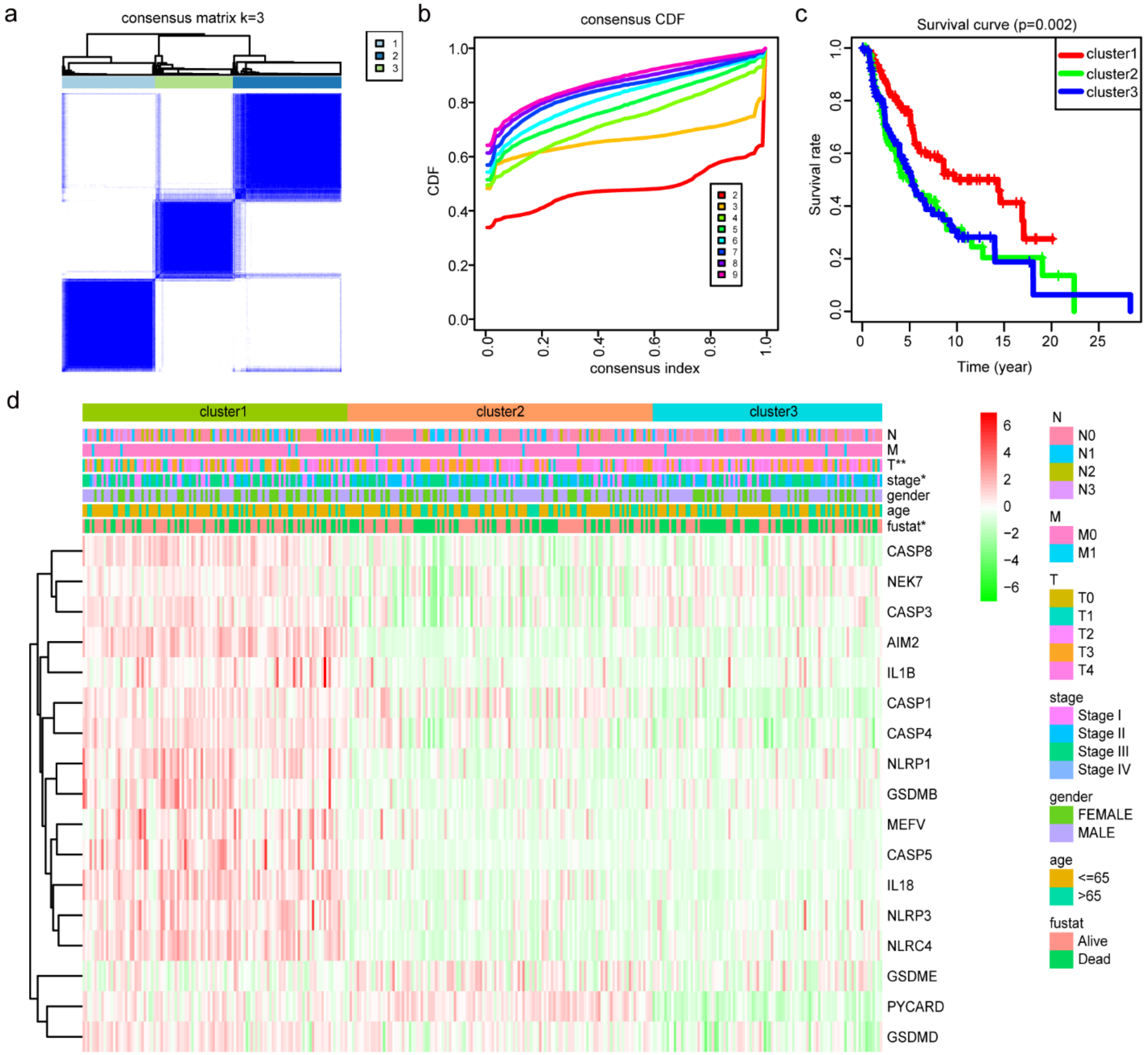

Supplementary Figure 7. The molecular classification using the expression of 17 pyroptotic factors in SKCM cohort. (a). Consensus clustering of cumulative distribution function (CDF) for k = 3; (b). Relative change in the area under the CDF curve for k = 2–9; (c). Kaplan–Meier overall survival (OS) curves of the in SKCM patients; (d). Heatmap and clinicopathological features of the three clusters defined by the consensus expression of the pyroptosis factors.

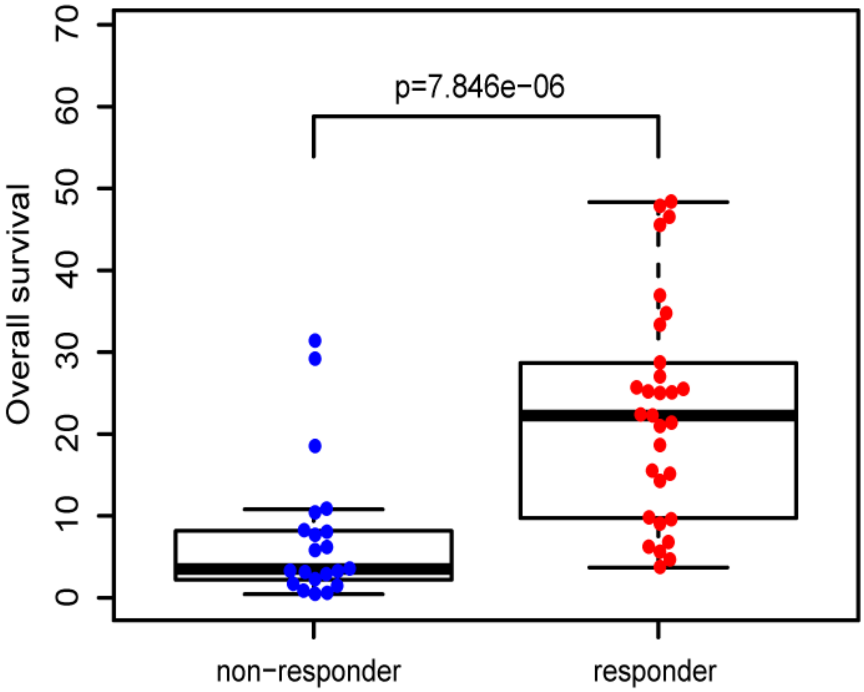

Supplementary Figure 8. The difference of overall survival between responders and non-responders with anti-PD-1 therapy in melanoma.

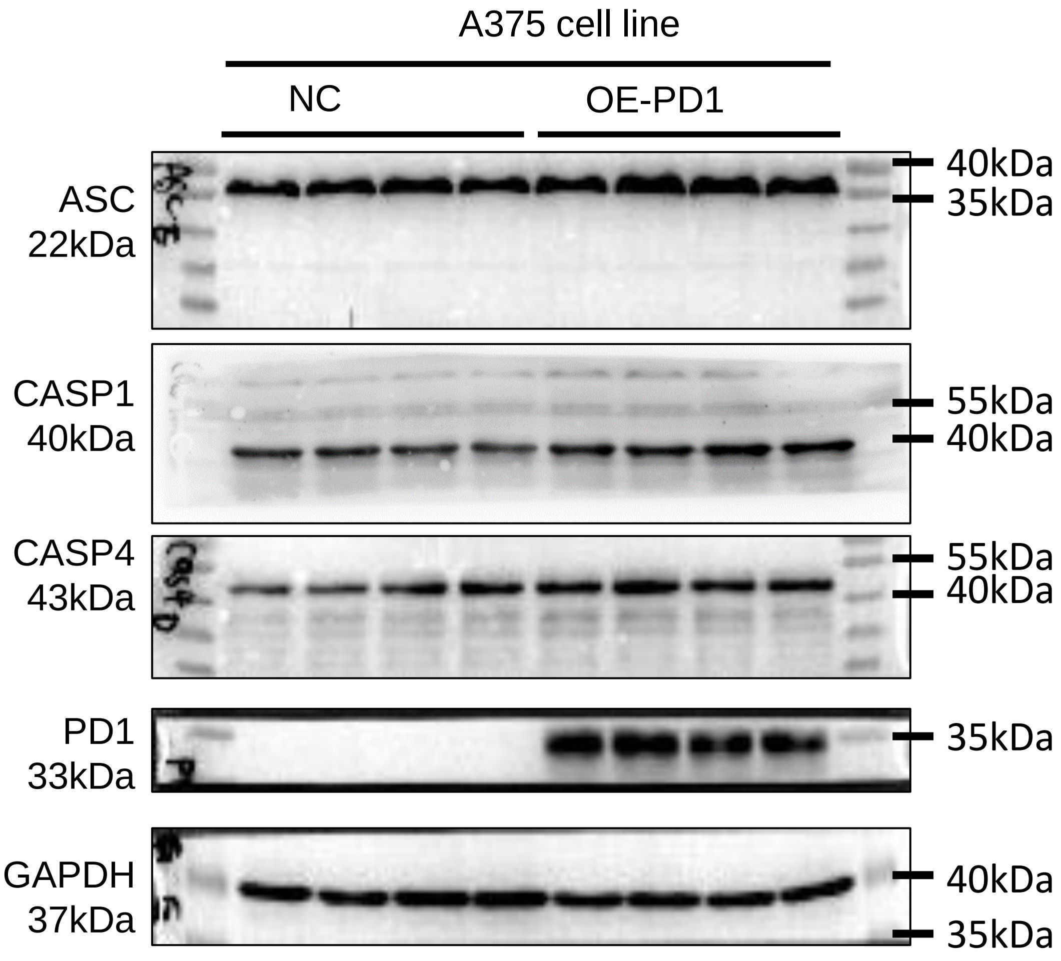

Supplementary Figure 9. Unedited gel for Figure 5g

Supplementary Table 1. The relative expression levels of pyroptosis-associated genes (PAGs) across 17 cancer types

cancertype	BLCA	BRCA	CHOL	COAD	ESCA	HNSC	KICH	KIRC	KIRP	LIHC	LUAD	LUSC	PRAD	READ	STAD	THCA	UCEC
CASP1	-0.054	-0.339	0.393	-0.130	0.752	0.203	-0.705	0.859	0.657	0.040	-0.393	-0.305	-0.473	-0.106	0.050	0.134	-0.137
CASP3	0.231	0.237	0.432	-0.102	0.248	0.147	0.087	0.251	0.387	0.407	0.299	0.363	0.017	-0.138	0.151	0.113	0.337
CASP4	0.116	-0.234	0.710	0.307	0.202	0.053	-0.298	0.566	0.492	0.108	0.061	-0.204	0.054	0.171	0.134	0.024	0.027
CASP5	1.956	0.656	0.949	-0.940	1.198	1.125	0.416	2.672	1.113	0.779	-1.076	-1.173	-0.177	-0.769	0.384	0.563	1.840
CASP8	0.338	0.062	1.011	0.111	0.426	0.458	-0.444	0.364	0.388	0.593	0.225	-0.024	0.168	0.191	0.507	0.053	0.281
NLRP3	-0.795	-0.527	0.774	-0.401	0.292	0.347	-0.492	1.047	0.660	-0.342	-0.506	-1.332	-0.346	-0.861	0.272	-0.070	-0.519
NLRP1	-0.368	-0.695	0.921	-0.352	0.433	0.520	-1.196	0.703	0.219	0.941	-0.284	-0.313	-0.468	-0.488	-0.103	0.068	-1.147
PYCARD	0.401	0.553	1.372	-0.067	0.706	0.261	-0.367	0.897	0.867	0.523	-0.174	-0.017	-0.346	0.106	0.210	0.454	0.241
NEK7	-0.194	-0.262	0.601	-0.215	0.085	0.115	-0.275	-0.057	-0.178	0.317	-0.278	-0.368	-0.245	-0.425	0.052	-0.131	-0.440
NLRC4	0.153	-0.048	0.495	-0.357	0.376	0.571	-0.255	1.376	0.681	-0.166	-1.180	-1.695	0.034	-0.391	0.828	0.337	0.089
MEFV	0.217	0.040	0.550	-0.378	0.704	1.437	-0.382	1.779	0.427	-0.430	-1.331	-1.452	-0.217	-0.229	0.536	-0.024	1.036
AIM2	0.793	1.116	1.716	0.123	1.500	2.119	-0.104	2.314	0.959	0.085	1.445	1.241	0.197	-0.298	0.685	-0.864	1.173
GSDMD	0.238	0.342	0.373	-0.048	0.426	0.408	-0.340	0.240	0.087	0.361	0.053	-0.067	-0.114	0.104	0.178	0.070	0.128
GSDMB	0.541	-0.149	0.317	-0.189	0.312	0.918	-0.881	1.378	1.307	0.797	0.950	0.610	0.554	0.004	0.444	0.576	0.539
GSDME	-0.182	-0.600	1.414	0.001	0.701	1.346	-1.732	-0.038	0.393	0.849	0.318	0.758	-0.540	-0.286	0.393	-0.068	-1.179
IL18	-0.079	0.562	1.621	-0.311	0.316	-0.132	0.775	0.322	0.512	0.059	-0.190	-0.197	-0.404	-0.339	0.164	0.429	0.631
IL1B	0.027	-0.309	0.630	0.688	0.826	0.608	-0.696	0.250	-0.024	-0.944	-0.612	-0.398	-0.368	0.213	0.108	-0.324	0.182

Supplementary Table 2. The RNA level of PD-L1 and proportion of TILs in 30 cancer types

ID	TIL	CD274	type
ACC	0.648	0.488	cold
BLCA	0.707	1.405	hot
BRCA	0.737	1.047	cold
CESC	0.733	1.961	hot
CHOL	0.683	0.882	cold
COAD	0.711	0.962	cold
ESCA	0.677	1.397	hot
GBM	0.679	1.292	hot
HNSC	0.744	1.942	hot
KICH	0.661	1.792	hot
KIRC	0.773	1.377	hot
KIRP	0.702	1.297	hot
LGG	0.648	0.686	cold
LIHC	0.732	0.500	cold
LUAD	0.733	1.946	hot
LUSC	0.725	2.171	hot
MESO	0.704	1.338	hot
OV	0.682	0.762	cold
PAAD	0.736	1.072	cold
PCPG	0.651	1.441	hot
PRAD	0.654	0.539	cold
READ	0.699	0.812	cold
SARC	0.718	1.016	cold
SKCM	0.758	1.203	hot
STAD	0.761	1.293	hot
TGCT	0.786	1.288	hot
THCA	0.712	1.628	hot
UCEC	0.700	0.662	cold
UCS	0.641	0.404	cold
UVM	0.649	0.692	cold

Supplementary Table 3. The mutation frequency of the pyroptosis-associated genes (PAGs) across 33 cancer types

CancerType	ACC	BLCA	BRCA	CESC	CHOL	COAD	DLBC	ESCA	GBM	HNSC	KICH	KIRC	KIRP	LAML	LGG	LIHC	LUAD	LUSC	MESO	OV	PAAD	PCPG	PRAD	READ	SARC	SKCM	STAD	TGCT	THCA	THYM	UCEC	UCS	UVM
CASP1	0.000	0.027	0.005	0.010	0.000	0.018	0.027	0.011	0.010	0.006	0.000	0.006	0.004	0.000	0.002	0.000	0.021	0.020	0.000	0.005	0.000	0.000	0.002	0.022	0.004	0.024	0.005	0.000	0.000	0.000	0.079	0.000	0.000
CASP3	0.000	0.010	0.002	0.014	0.000	0.020	0.000	0.000	0.003	0.006	0.000	0.000	0.000	0.007	0.000	0.005	0.005	0.000	0.000	0.000	0.000	0.000	0.000	0.015	0.000	0.019	0.005	0.000	0.002	0.000	0.053	0.000	0.000
CASP4	0.000	0.007	0.002	0.007	0.000	0.015	0.000	0.000	0.005	0.004	0.000	0.000	0.007	0.000	0.004	0.008	0.012	0.014	0.013	0.007	0.006	0.000	0.000	0.022	0.008	0.017	0.025	0.000	0.002	0.000	0.045	0.018	0.000
CASP5	0.000	0.022	0.004	0.017	0.000	0.043	0.000	0.011	0.003	0.008	0.000	0.000	0.004	0.000	0.000	0.003	0.025	0.018	0.000	0.005	0.006	0.000	0.000	0.022	0.004	0.032	0.042	0.000	0.000	0.000	0.078	0.000	0.000
CASP8	0.011	0.027	0.018	0.048	0.020	0.050	0.000	0.016	0.000	0.097	0.000	0.006	0.004	0.000	0.004	0.008	0.007	0.022	0.000	0.007	0.006	0.000	0.002	0.029	0.004	0.026	0.046	0.007	0.000	0.000	0.115	0.018	0.000
NLRP3	0.043	0.032	0.013	0.017	0.020	0.065	0.000	0.016	0.036	0.036	0.015	0.006	0.007	0.007	0.006	0.022	0.146	0.116	0.000	0.028	0.013	0.000	0.008	0.029	0.008	0.203	0.046	0.000	0.002	0.000	0.108	0.000	0.013
NLRP1	0.033	0.027	0.013	0.038	0.000	0.090	0.027	0.027	0.010	0.018	0.015	0.003	0.028	0.007	0.006	0.011	0.037	0.029	0.000	0.002	0.006	0.000	0.004	0.029	0.013	0.156	0.037	0.000	0.000	0.000	0.121	0.018	0.000
PYCARD	0.011	0.010	0.001	0.000	0.000	0.003	0.000	0.000	0.008	0.006	0.000	0.009	0.000	0.000	0.002	0.003	0.002	0.004	0.000	0.000	0.000	0.000	0.002	0.007	0.000	0.002	0.014	0.000	0.000	0.000	0.011	0.000	0.000
NEK7	0.000	0.010	0.001	0.014	0.000	0.018	0.027	0.005	0.003	0.000	0.000	0.000	0.007	0.000	0.000	0.003	0.005	0.006	0.000	0.000	0.006	0.000	0.002	0.015	0.000	0.013	0.023	0.000	0.000	0.000	0.057	0.018	0.000
NLRC4	0.011	0.024	0.016	0.024	0.000	0.035	0.000	0.033	0.003	0.022	0.000	0.009	0.007	0.015	0.008	0.011	0.027	0.031	0.000	0.005	0.006	0.000	0.000	0.029	0.000	0.081	0.025	0.007	0.002	0.008	0.095	0.018	0.000
MEFV	0.043	0.024	0.010	0.021	0.000	0.063	0.000	0.005	0.013	0.016	0.000	0.006	0.007	0.007	0.000	0.016	0.037	0.039	0.000	0.011	0.032	0.006	0.006	0.037	0.017	0.094	0.035	0.000	0.002	0.000	0.074	0.000	0.000
AIM2	0.000	0.015	0.004	0.010	0.000	0.020	0.000	0.005	0.003	0.004	0.000	0.000	0.004	0.000	0.002	0.008	0.018	0.022	0.000	0.011	0.000	0.000	0.006	0.022	0.000	0.030	0.018	0.000	0.000	0.000	0.045	0.000	0.000
GSDMD	0.000	0.010	0.000	0.003	0.020	0.015	0.000	0.011	0.003	0.012	0.000	0.003	0.007	0.000	0.006	0.005	0.007	0.010	0.000	0.000	0.006	0.000	0.002	0.007	0.004	0.009	0.002	0.000	0.000	0.000	0.034	0.000	0.000
GSDMB	0.000	0.002	0.001	0.010	0.000	0.010	0.027	0.000	0.008	0.002	0.000	0.003	0.000	0.000	0.002	0.005	0.005	0.008	0.000	0.005	0.006	0.000	0.004	0.007	0.000	0.034	0.012	0.000	0.000	0.000	0.026	0.000	0.000
GSDME	0.000	0.000	0.000	0.000	0.000	0.000	0.000	0.000	0.000	0.000	0.000	0.000	0.000	0.000	0.000	0.000	0.000	0.000	0.000	0.000	0.000	0.000	0.000	0.000	0.000	0.000	0.000	0.000	0.000	0.000	0.000	0.000	0.000
IL1B	0.000	0.000	0.000	0.000	0.000	0.000	0.000	0.000	0.000	0.000	0.000	0.000	0.000	0.000	0.000	0.000	0.000	0.000	0.000	0.000	0.000	0.000	0.000	0.000	0.000	0.000	0.000	0.000	0.000	0.000	0.000	0.000	0.000
IL18	0.011	0.000	0.002	0.000	0.000	0.013	0.000	0.000	0.003	0.000	0.000	0.000	0.004	0.000	0.004	0.003	0.000	0.000	0.000	0.000	0.000	0.000	0.002	0.000	0.000	0.011	0.002	0.000	0.000	0.008	0.026	0.000	0.000

Supplementary Table 4. The copy number amplification frequency of the pyroptosis-associated genes (PAGs) across 33 cancer types

CancerType	ACC	BLCA	BRCA	CESC	CHOL	COAD	DLBC	ESCA	GBM	HNSC	KICH	KIRC	KIRP	LAML	LGG	LIHC	LUAD	LUSC	MESO	OV	PAAD	PCPG	PRAD	READ	SARC	SKCM	STAD	TGCT	THCA	THYM	UCEC	UCS	UVM
CASP1	0.000	0.041	0.034	0.027	0.000	0.012	0.021	0.054	0.002	0.029	0.000	0.002	0.000	0.010	0.008	0.013	0.045	0.038	0.011	0.158	0.011	0.000	0.006	0.024	0.056	0.017	0.011	0.006	0.000	0.000	0.020	0.089	0.000
CASP3	0.044	0.034	0.045	0.020	0.000	0.006	0.021	0.092	0.005	0.019	0.000	0.002	0.007	0.000	0.004	0.026	0.018	0.040	0.000	0.085	0.000	0.006	0.018	0.024	0.052	0.030	0.018	0.026	0.002	0.000	0.018	0.054	0.013
CASP4	0.000	0.041	0.034	0.027	0.000	0.012	0.021	0.054	0.002	0.029	0.000	0.002	0.000	0.010	0.008	0.013	0.045	0.040	0.011	0.161	0.005	0.000	0.006	0.024	0.056	0.017	0.014	0.006	0.000	0.000	0.020	0.089	0.000
CASP5	0.000	0.041	0.033	0.027	0.000	0.012	0.021	0.054	0.002	0.029	0.000	0.002	0.000	0.010	0.008	0.013	0.045	0.038	0.011	0.160	0.011	0.000	0.006	0.024	0.060	0.017	0.014	0.006	0.000	0.000	0.020	0.089	0.000
CASP8	0.033	0.017	0.043	0.040	0.000	0.014	0.000	0.070	0.002	0.042	0.000	0.012	0.010	0.000	0.002	0.042	0.041	0.061	0.000	0.145	0.032	0.000	0.008	0.018	0.052	0.019	0.039	0.032	0.002	0.000	0.027	0.036	0.000
NLRP3	0.067	0.075	0.161	0.057	0.056	0.026	0.042	0.124	0.013	0.053	0.000	0.007	0.010	0.010	0.011	0.100	0.097	0.092	0.034	0.237	0.027	0.006	0.014	0.035	0.049	0.095	0.091	0.019	0.004	0.008	0.068	0.179	0.050
NLRP1	0.022	0.027	0.026	0.003	0.000	0.008	0.000	0.022	0.002	0.008	0.000	0.002	0.000	0.000	0.000	0.003	0.011	0.013	0.000	0.106	0.005	0.000	0.004	0.000	0.067	0.023	0.018	0.000	0.000	0.000	0.029	0.036	0.000
PYCARD	0.000	0.075	0.057	0.013	0.028	0.038	0.042	0.043	0.003	0.011	0.000	0.002	0.010	0.000	0.008	0.024	0.070	0.078	0.000	0.152	0.000	0.006	0.002	0.035	0.045	0.013	0.045	0.013	0.000	0.000	0.071	0.179	0.000
NEK7	0.044	0.048	0.126	0.027	0.056	0.020	0.167	0.054	0.013	0.027	0.000	0.010	0.000	0.010	0.008	0.098	0.092	0.046	0.034	0.155	0.022	0.012	0.010	0.041	0.049	0.081	0.030	0.019	0.004	0.008	0.046	0.054	0.038
MEFV	0.044	0.012	0.062	0.064	0.028	0.024	0.021	0.043	0.011	0.029	0.000	0.003	0.003	0.000	0.004	0.021	0.040	0.013	0.034	0.090	0.005	0.000	0.016	0.024	0.064	0.038	0.032	0.000	0.000	0.000	0.020	0.071	0.000
AIM2	0.044	0.186	0.132	0.064	0.139	0.026	0.146	0.103	0.013	0.023	0.030	0.020	0.000	0.000	0.015	0.140	0.135	0.116	0.034	0.219	0.049	0.024	0.014	0.047	0.142	0.072	0.066	0.051	0.006	0.008	0.066	0.161	0.000
GSDMD	0.022	0.111	0.165	0.111	0.167	0.057	0.104	0.178	0.013	0.110	0.000	0.020	0.007	0.005	0.064	0.140	0.153	0.240	0.069	0.485	0.092	0.006	0.026	0.106	0.109	0.104	0.116	0.038	0.000	0.008	0.064	0.089	0.188
GSDMB	0.022	0.096	0.137	0.030	0.028	0.061	0.021	0.205	0.003	0.029	0.000	0.000	0.010	0.000	0.002	0.018	0.058	0.061	0.000	0.079	0.076	0.000	0.008	0.071	0.037	0.017	0.170	0.006	0.000	0.000	0.077	0.179	0.000
GSDME	0.000	0.041	0.042	0.010	0.000	0.030	0.000	0.076	0.018	0.017	0.000	0.007	0.000	0.000	0.004	0.026	0.059	0.069	0.000	0.098	0.016	0.000	0.016	0.018	0.075	0.036	0.034	0.038	0.002	0.000	0.033	0.036	0.000
IL18	0.000	0.024	0.022	0.003	0.000	0.006	0.063	0.032	0.003	0.010	0.000	0.000	0.000	0.010	0.008	0.013	0.029	0.029	0.023	0.105	0.011	0.000	0.002	0.018	0.019	0.004	0.014	0.000	0.000	0.000	0.013	0.071	0.000
NLRC4	0.011	0.043	0.041	0.034	0.000	0.014	0.000	0.038	0.005	0.015	0.000	0.000	0.007	0.000	0.004	0.042	0.040	0.067	0.023	0.132	0.000	0.000	0.012	0.006	0.045	0.008	0.018	0.071	0.002	0.000	0.053	0.143	0.013
IL1B	0.022	0.041	0.029	0.010	0.000	0.010	0.000	0.108	0.000	0.051	0.000	0.002	0.000	0.000	0.002	0.021	0.034	0.053	0.000	0.090	0.005	0.000	0.002	0.000	0.097	0.008	0.045	0.006	0.002	0.000	0.051	0.089	0.000

Supplementary Table 5. The copy number deletion frequency of the pyroptosis-associated genes (PAGs) across 33 cancer types

CancerType	ACC	BLCA	BRCA	CESC	CHOL	COAD	DLBC	ESCA	GBM	HNSC	KICH	KIRC	KIRP	LAML	LGG	LIHC	LUAD	LUSC	MESO	OV	PAAD	PCPG	PRAD	READ	SARC	SKCM	STAD	TGCT	THCA	THYM	UCEC	UCS	UVM
CASP1	0.022	0.063	0.131	0.114	0.056	0.024	0.000	0.038	0.008	0.101	0.015	0.010	0.026	0.000	0.011	0.045	0.049	0.050	0.069	0.102	0.016	0.041	0.052	0.024	0.064	0.110	0.041	0.038	0.000	0.008	0.046	0.089	0.013
CASP3	0.133	0.075	0.062	0.074	0.250	0.093	0.104	0.081	0.043	0.093	0.030	0.022	0.007	0.000	0.069	0.095	0.068	0.109	0.046	0.181	0.049	0.036	0.028	0.059	0.187	0.055	0.116	0.006	0.002	0.008	0.047	0.054	0.000
CASP4	0.022	0.063	0.131	0.114	0.056	0.022	0.000	0.038	0.011	0.099	0.015	0.008	0.026	0.000	0.011	0.045	0.049	0.048	0.069	0.102	0.016	0.036	0.052	0.024	0.064	0.110	0.039	0.038	0.000	0.008	0.046	0.089	0.013
CASP5	0.022	0.063	0.131	0.114	0.056	0.028	0.000	0.038	0.010	0.101	0.015	0.010	0.026	0.000	0.011	0.045	0.049	0.052	0.069	0.103	0.016	0.041	0.052	0.024	0.060	0.110	0.041	0.038	0.000	0.008	0.046	0.089	0.013
CASP8	0.000	0.118	0.028	0.135	0.000	0.012	0.021	0.092	0.003	0.101	0.015	0.024	0.007	0.005	0.009	0.008	0.027	0.086	0.057	0.094	0.000	0.000	0.012	0.000	0.060	0.025	0.030	0.006	0.002	0.000	0.016	0.036	0.013
NLRP3	0.044	0.046	0.030	0.020	0.028	0.010	0.146	0.027	0.025	0.011	0.000	0.015	0.013	0.000	0.036	0.024	0.016	0.031	0.046	0.095	0.005	0.030	0.028	0.012	0.150	0.019	0.011	0.013	0.000	0.008	0.013	0.000	0.013
NLRP1	0.033	0.022	0.030	0.024	0.056	0.008	0.063	0.027	0.032	0.023	0.000	0.003	0.007	0.010	0.002	0.071	0.045	0.029	0.023	0.027	0.005	0.012	0.090	0.006	0.187	0.021	0.014	0.032	0.004	0.000	0.029	0.071	0.000
PYCARD	0.000	0.014	0.011	0.020	0.000	0.000	0.000	0.000	0.005	0.015	0.000	0.000	0.000	0.010	0.011	0.000	0.018	0.010	0.000	0.023	0.005	0.000	0.010	0.000	0.015	0.004	0.002	0.013	0.000	0.000	0.002	0.036	0.000
NEK7	0.011	0.060	0.013	0.003	0.028	0.008	0.000	0.054	0.011	0.004	0.000	0.000	0.003	0.005	0.008	0.016	0.011	0.027	0.000	0.061	0.005	0.006	0.014	0.018	0.082	0.019	0.016	0.000	0.000	0.000	0.013	0.018	0.000
MEFV	0.011	0.128	0.018	0.007	0.028	0.030	0.021	0.065	0.005	0.021	0.000	0.000	0.000	0.000	0.000	0.018	0.018	0.055	0.023	0.097	0.011	0.000	0.002	0.029	0.019	0.015	0.048	0.000	0.000	0.000	0.026	0.018	0.000
AIM2	0.000	0.002	0.023	0.003	0.056	0.002	0.000	0.011	0.002	0.006	0.000	0.000	0.003	0.000	0.000	0.011	0.004	0.004	0.000	0.027	0.000	0.006	0.038	0.000	0.019	0.013	0.007	0.000	0.000	0.000	0.005	0.000	0.000
GSDMD	0.000	0.039	0.052	0.020	0.000	0.038	0.000	0.049	0.016	0.013	0.000	0.000	0.000	0.000	0.039	0.024	0.025	0.025	0.034	0.039	0.005	0.018	0.040	0.029	0.082	0.019	0.039	0.064	0.004	0.000	0.029	0.018	0.000
GSDMB	0.078	0.017	0.043	0.017	0.028	0.004	0.000	0.022	0.030	0.011	0.000	0.000	0.000	0.010	0.013	0.042	0.016	0.036	0.023	0.069	0.000	0.059	0.032	0.000	0.101	0.023	0.014	0.013	0.000	0.016	0.044	0.071	0.025
GSDME	0.000	0.024	0.027	0.007	0.000	0.004	0.063	0.027	0.025	0.017	0.000	0.003	0.000	0.005	0.006	0.005	0.007	0.019	0.000	0.103	0.000	0.000	0.008	0.006	0.026	0.013	0.020	0.013	0.002	0.000	0.018	0.036	0.000
IL18	0.033	0.077	0.155	0.162	0.111	0.028	0.000	0.054	0.008	0.116	0.015	0.010	0.026	0.000	0.004	0.058	0.065	0.067	0.115	0.121	0.005	0.041	0.072	0.035	0.109	0.159	0.061	0.077	0.000	0.008	0.042	0.054	0.025
NLRC4	0.022	0.019	0.019	0.010	0.028	0.004	0.063	0.043	0.008	0.013	0.000	0.005	0.007	0.000	0.002	0.005	0.022	0.015	0.057	0.048	0.032	0.006	0.034	0.047	0.045	0.019	0.048	0.013	0.002	0.000	0.016	0.000	0.000
IL1B	0.011	0.017	0.027	0.013	0.028	0.002	0.063	0.022	0.008	0.011	0.000	0.003	0.003	0.000	0.002	0.016	0.007	0.019	0.000	0.095	0.005	0.000	0.024	0.000	0.007	0.011	0.016	0.013	0.000	0.000	0.013	0.018	0.000

Supplementary Table 6. Patient characteristics stratified by risk score in the skin cutaneous melanoma (SKCM) cohort

	level	risk score	high	low	p
n		339	169	170	
fustat (%)	Alive	155 (45.7)	87 (51.5)	68 (40.0)	0.044
	Dead	184 (54.3)	82 (48.5)	102 (60.0)	
age (%)	<=65	222 (65.5)	99 (58.6)	123 (72.4)	0.011
	>65	117 (34.5)	70 (41.4)	47 (27.6)	
gender (%)	FEMALE	130 (38.3)	63 (37.3)	67 (39.4)	0.77
	MALE	209 (61.7)	106 (62.7)	103 (60.6)	
stage (%)	Stage I	70 (20.6)	25 (14.8)	45 (26.5)	<0.001
	Stage II	115 (33.9)	77 (45.6)	38 (22.4)	
	Stage III	143 (42.2)	63 (37.3)	80 (47.1)	
	Stage IV	11 (3.2)	4 (2.4)	7 (4.1)	
T (%)	T0	23 (6.8)	3 (1.8)	20 (11.8)	<0.001
	T1	36 (10.6)	11 (6.5)	25 (14.7)	
	T2	69 (20.4)	30 (17.8)	39 (22.9)	
	T3	83 (24.5)	40 (23.7)	43 (25.3)	
	T4	128 (37.8)	85 (50.3)	43 (25.3)	
N (%)	N0	193 (56.9)	106 (62.7)	87 (51.2)	0.071
	N1	65 (19.2)	24 (14.2)	41 (24.1)	
	N2	41 (12.1)	18 (10.7)	23 (13.5)	
	N3	40 (11.8)	21 (12.4)	19 (11.2)	
M (%)	M0	328 (96.8)	165 (97.6)	163 (95.9)	0.542
	M1	11 (3.2)	4 (2.4)	7 (4.1)	

Supplementary Table 7. Patient characteristics stratified based on clustering type in the skin cutaneous melanoma (SKCM) cohort

	level	cluster	cluster1	cluster2	cluster3	p
n		338	112	129	97	
fustat (%)	Alive	155 (45.9)	44 (39.3)	56 (43.4)	55 (56.7)	0.033
	Dead	183 (54.1)	68 (60.7)	73 (56.6)	42 (43.3)	
age (%)	<=65	222 (65.7)	81 (72.3)	81 (62.8)	60 (61.9)	0.192
	>65	116 (34.3)	31 (27.7)	48 (37.2)	37 (38.1)	
gender (%)	FEMALE	130 (38.5)	47 (42.0)	47 (36.4)	36 (37.1)	0.644
	MALE	208 (61.5)	65 (58.0)	82 (63.6)	61 (62.9)	
stage (%)	Stage I	69 (20.4)	31 (27.7)	21 (16.3)	17 (17.5)	0.01
	Stage II	115 (34.0)	22 (19.6)	55 (42.6)	38 (39.2)	
	Stage III	143 (42.3)	55 (49.1)	49 (38.0)	39 (40.2)	
	Stage IV	11 (3.3)	4 (3.6)	4 (3.1)	3 (3.1)	
T (%)	T0	23 (6.8)	13 (11.6)	6 (4.7)	4 (4.1)	0.001
	T1	35 (10.4)	20 (17.9)	9 (7.0)	6 (6.2)	
	T2	69 (20.4)	23 (20.5)	20 (15.5)	26 (26.8)	
	T3	83 (24.6)	27 (24.1)	33 (25.6)	23 (23.7)	
	T4	128 (37.9)	29 (25.9)	61 (47.3)	38 (39.2)	
N (%)	N0	192 (56.8)	57 (50.9)	78 (60.5)	57 (58.8)	0.187
	N1	65 (19.2)	24 (21.4)	28 (21.7)	13 (13.4)	
	N2	41 (12.1)	18 (16.1)	12 (9.3)	11 (11.3)	
	N3	40 (11.8)	13 (11.6)	11 (8.5)	16 (16.5)	
M (%)	M0	327 (96.7)	108 (96.4)	125 (96.9)	94 (96.9)	1
	M1	11 (3.3)	4 (3.6)	4 (3.1)	3 (3.1)	

Supplementary Table 8.The case numbers of data analysis of 33 cancer types from TCGA pan-cancer project

Cancers	Abbreviation	Numbers of Normal Samples	Numbers of Cancer Samples	Number of samples with CNV	Number of samples with mutation
Adrenocortical Carcinoma	ACC	0	79	90	92
Bladder Urothelial Carcinoma	BLCA	19	411	415	412
Breast Invasive Carcinoma	BRCA	113	1104	1106	986
Cervical Squamous Cell Carcinoma and Endocervical Adenocarcinoma	CESC	3	306	297	289
Cholangiocarcinoma	CHOL	9	36	36	51
Colon Adenocarcinoma	COAD	41	471	506	399
Lymphoid Neoplasm Diffuse Large B-cell Lymphoma	DLBC	0	48	48	37
Esophageal Carcinoma	ESCA	11	162	185	184
Glioblastoma Multiforme	GBM	5	168	628	390
Head and Neck Squamous Carcinoma	HNSC	44	502	526	506
Kidney Chromophobe	KICH	24	65	66	66
Kidney Renal Clear Cell Carcinoma	KIRC	72	535	589	336
Kidney Renal Papillary Cell Carcinoma	KIRP	32	289	303	281
Acute Myeloid Leukemia	LAML	0	151	194	134
Brain Lower Grade Glioma	LGG	0	529	533	506
Liver Hepatocellular Carcinoma	LIHC	50	374	379	364
Lung Adenocarcinoma	LUAD	59	526	555	561
Lung Squamous Cell Carcinoma	LUSC	49	501	524	491
Mesothelioma	MESO	0	86	87	80
Ovarian Serous Cystadenocarcinoma	OV	0	379	620	436
Pancreatic Adenocarcinoma	PAAD	4	178	185	158
Pheochromocytoma and Paraganglioma	PCPG	3	183	169	178
Prostate Adenocarcinoma	PRAD	52	499	502	484
Rectum Adenocarcinoma	READ	10	167	170	136
Sarcoma	SARC	2	263	267	237
Skin Cutaneous Melanoma	SKCM	0	471	472	467
Stomach Adenocarcinoma	STAD	32	375	440	433
Testicular Germ Cell Tumors	TGCT	0	156	156	145
Thyroid Carcinoma	THCA	58	510	512	487
Thymoma	THYM	2	119	124	122
Uterine Corpus Endometrial Carcinoma	UCEC	35	548	548	529
Uterine Carcinosarcoma	UCS	0	56	56	57
Uveal Melanoma	UVM	0	80	80	80

Supplementary Table 9. Quantitative RT-PCR primers for this study.

Gene symbol	Forward primer	Reverse primer
MIF	AGAACCGCTCCTACAGCAAGCT	GGAGTTGTTCCAGCCCACATTG
TNFα	CTCTTCTGCCTGCTGCACTTTG	ATGGGCTACAGGCTTGTCACTC
IL6	AGACAGCCACTCACCTCTTCAG	TTCTGCCAGTGCCTCTTTGCTG
IL10	TCTCCGAGATGCCTTCAGCAGA	TCAGACAAGGCTTGGCAACCCA
IL12A	TGCCTTCACCACTCCCAAAACC	CAATCTCTTCAGAAGTGCAAGGG
IL17A	CGGACTGTGATGGTCAACCTGA	GCACTTTGCCTCCCAGATCACA
IL23A	GAGCCTTCTCTGCTCCCTGATA	GACTGAGGCTTGGAATCTGCTG
TGFB1	TACCTGAACCCGTGTTGCTCTC	GTTGCTGAGGTATCGCCAGGAA
PD1	AAGGCGCAGATCAAAGAGAGCC	CAACCACCAGGGTTTGGAACTG
PDL1	TGCCGACTACAAGCGAATTACTG	CTGCTTGTCCAGATGACTTCGG
LAG3	GCAGTGTACTTCACAGAGCTGTC	AAGCCAAAGGCTCCAGTCACCA
IDO1	GCCTGATCTCATAGAGTCTGGC	TGCATCCCAGAACTAGACGTGC
TNFRSF8	GGGTGATCCTGGTGTTGGTT	CCTGGGTCTGGAATCCACAAG
PDL2	AGGGAAGTGAACAGTGCTATCTG	GCCAGGTGTTGGCTAGTCTT
TIM3	GACTCTAGCAGACAGTGGGATC	GGTGGTAAGCATCCTTGGAAAGG
CTLA4	TACCCACCGCCATACTACCT	TGGGCACGGTTCTGGATCAAT
VTCN1	CAGATCCTCTTCTGGAGCATA	CCATCCTCCCCAATGTTCC
GPR146	ACGCCACACTATCTGATCCTGC	GAGAAGTGGTGTCACAAAGCTGC
SELP	AACCTACCACCCCTTCCTG	AGATGCCACCTGCTTTCCA
AHR	GTCGTCTAAGGTGTCTGCTGGA	CGCAAACAAAGCCAACTGAGGTG
DDX4	GTGTCTGGACATGATGCACCAC	GCAAGCCATCAAATCTCGTCCTG
IFIT1	GCCTTGCTGAAGTGTGGAGGAA	ATCCAGGCGATAGGCAGAGATC
IFIT2	GGAGCAGATTCTGAGGCTTTGC	GGATGAGGCTTCCAGACTCCAA
IFIT3	CCTGGAATGCTTACGGCAAGCT	GAGCATCTGAGAGTCTGCCCAA
IRF7	CCACGCTATACCATCTACCTGG	GCTGCTATCCAGGGAAGACACA
ISG20	ACACGTCCACTGACAGGCTGTT	ATCTTCCACCGAGCTGTGTCCA
MX1	GGCTGTTTACCAGACTCCGACA	CACAAAGCCTGGCAGCTCTCTA
MX2	AAAAGCAGCCCTGTGAGGCATG	GTGATCTCCAGGCTGATGAGCT
RSAD2	CCAGTGCAACTACAAATGCGGC	CGGTCTTGAAGAAATGGCTCTCC
TNFSF10	TGGCAACTCCGTCAGCTCGTTA	AGCTGCTACTCTCTGAGGACCT
