# Supplementary material for: Pyroptosis correlates with tumor immunity and prognosis
Source: Commun Biol. 2022 Sep 6;5:917. doi: 10.1038/s42003-022-03806-x (PMC9448722; doi:10.1038/s42003-022-03806-x)
Supplement: Supplementary file 4 — Reporting Summary [file 42003_2022_3806_MOESM4_ESM.pdf]

nature portfolio

Corresponding author(s): Cui Wei,COMMSBIO-21-1193C

Last updated by author(s): Jul 12, 2022

Reporting Summary

Nature Portfolio wishes to improve the reproducibility of the work that we publish. This form provides structure for consistency and transparency in reporting. For further information on Nature Portfolio policies, see our Editorial Policies and the Editorial Policy Checklist.

Statistics

For all statistical analyses, confirm that the following items are present in the figure legend, table legend, main text, or Methods section.

n/a	Confirmed	
☐	☒	The exact sample size (*n*) for each experimental group/condition, given as a discrete number and unit of measurement
☐	☒	A statement on whether measurements were taken from distinct samples or whether the same sample was measured repeatedly
☐	☒	The statistical test(s) used AND whether they are one- or two-sided<br>*Only common tests should be described solely by name; describe more complex techniques in the Methods section.*
☐	☒	A description of all covariates tested
☐	☒	A description of any assumptions or corrections, such as tests of normality and adjustment for multiple comparisons
☐	☒	A full description of the statistical parameters including central tendency (e.g. means) or other basic estimates (e.g. regression coefficient) AND variation (e.g. standard deviation) or associated estimates of uncertainty (e.g. confidence intervals)
☐	☒	For null hypothesis testing, the test statistic (e.g. *F*, *t*, *r*) with confidence intervals, effect sizes, degrees of freedom and *P* value noted<br>*Give P values as exact values whenever suitable.*
☒	☐	For Bayesian analysis, information on the choice of priors and Markov chain Monte Carlo settings
☒	☐	For hierarchical and complex designs, identification of the appropriate level for tests and full reporting of outcomes
☒	☐	Estimates of effect sizes (e.g. Cohen's *d*, Pearson's *r*), indicating how they were calculated

Our web collection on statistics for biologists contains articles on many of the points above.

Software and code

Policy information about availability of computer code

Data collection	The genomic,transcriptomic,and clinical data of 33 cancer types were downloaded from the UCSC Xena browser (http://xena.ucsc.edu/).<br>Software and resources used for the analyses are described in each method section.
Data analysis	the single-sample gene set enrichment analysis (ssGSEA) was used and conducted with the GSVA R package.<br>The Spearman correlation analysis were visualized using the corrplot package (https://github.com/taiyun/corrplot).<br>The hierarchical clustering analysis:the"hclust"function using R software (version 3.6.2,www.r-project.org)<br>Estimation of immune-cell type fractions:CIBERSORT algorithm<br>Estimation of the proportion of tumor-infiltrating cells:ESTIMATE package (https://R-Forge.R-project.org/projects/estimate/)<br>Consensus clustering approach:ConsensusClusterPlus package<br>The least absolute shrinkage and selection operator (LASSO)regression:the "glmnet"package.<br>The primers for RT qPCR are shown in Table S7.<br>Unprocessed images of all immunoblots can be obtained when request

For manuscripts utilizing custom algorithms or software that are central to the research but not yet described in published literature, software must be made available to editors and reviewers. We strongly encourage code deposition in a community repository (e.g. GitHub). See the Nature Portfolio guidelines for submitting code & software for further information.

Data

Policy information about availability of data

All manuscripts must include a data availability statement. This statement should provide the following information, where applicable:

- Accession codes, unique identifiers, or web links for publicly available datasets
- A description of any restrictions on data availability
- For clinical datasets or third party data, please ensure that the statement adheres to our policy

The genomic, transcriptomic, and clinical data of 33 cancer types were downloaded from the UCSC Xena browser (http://xena.ucsc.edu/). Software and resources used for the analyses are described in each method section. All other datasets generated during in this study are available from the corresponding authors upon reasonable request

Human research participants

Policy information about studies involving human research participants and Sex and Gender in Research.

Reporting on sex and gender	NA
Population characteristics	NA
Recruitment	NA
Ethics oversight	NA

Note that full information on the approval of the study protocol must also be provided in the manuscript.

Field-specific reporting

Please select the one below that is the best fit for your research. If you are not sure, read the appropriate sections before making your selection.

☒ Life sciences ☐ Behavioural & social sciences ☐ Ecological, evolutionary & environmental sciences

For a reference copy of the document with all sections, see nature.com/documents/nr-reporting-summary-flat.pdf

Life sciences study design

All studies must disclose on these points even when the disclosure is negative.

Sample size	No statistical methods were used to predetermine sample size.For bioinformatics analysis we included 33 cancer types from TCGA pan-cancer project and the specific sample size for different analysis can be found in supplementary table 1.For experiments we included at least3 independent replicates based on previous experience and the standard practices of the field.
Data exclusions	17 cancer types were sorted for the differential gene expression between cancer and normal,after excluding the case numbers were less than10 in each cancer type(Figure1).Since the high proportion of immune cells of Acute Myeloid Leukemia (LAML),Lymphoid Neoplasm DiffuseLarge B-cell Lymphoma(DLBC),and Thymoma(THYM),these cancers were excluded for clustering analysis and immune correlation analysis(Figure 1C-E and Figure 2).
Replication	All experiments were performed in at least biological triplicate with similar results.
Randomization	Randomization was not required for the type of in vitro data we have reported in this study
Blinding	No blinding was used in this study

Reporting for specific materials, systems and methods

We require information from authors about some types of materials, experimental systems and methods used in many studies. Here, indicate whether each material, system or method listed is relevant to your study. If you are not sure if a list item applies to your research, read the appropriate section before selecting a response.

Materials & experimental systems

n/a	Involved in the study
☐	☒ Antibodies
☒	☐ Eukaryotic cell lines
☒	☐ Palaeontology and archaeology
☒	☐ Animals and other organisms
☒	☐ Clinical data
☒	☐ Dual use research of concern

Methods

n/a	Involved in the study
☒	☐ ChIP-seq
☒	☐ Flow cytometry
☒	☐ MRI-based neuroimaging

Antibodies

Antibodies used	Anti-ASC (ab155970;Abcam);anti-caspase-1(ab207802;Abcam);anti-caspase-4 (ab238124;Abcam);anti-GSDMD(66387-1-Ig;Proteintech);anti-PD1 (ab52587;Abcam);anti-GAPDH(ab8245;Abcam).Anti-rabbit-HRP(#7074,Cell Signaling Technology)and anti-mouse-HRP(#7076,Cell Signaling Technology)
Validation	Antibodies were all validated by commercial source.No homemade or previously unpublished antibodies were used in this study.Allantibodies for proteins included in this study were validated using western blotting.
